# The tiger beetles (Coleoptera, Cicindelidae) of the southern Levant and adjacent territories: from cybertaxonomy to conservation biology

**DOI:** 10.3897/zookeys.734.21989

**Published:** 2018-02-05

**Authors:** Thorsten Assmann, Estève Boutaud, Jörn Buse, Jörg Gebert, Claudia Drees, Ariel-Leib-Leonid Friedman, Fares Khoury, Tamar Marcus, Eylon Orbach, Ittai Renan, Constantin Schmidt, Pascale Zumstein

**Affiliations:** 1 Institute of Ecology, Leuphana University Lüneburg, Universitätsallee 1, D-21335 Lüneburg, Germany; 2 Ecosystem Monitoring, Research and Wildlife Conservation (SB 23 Invertebrates and Biodiversity), Black Forest National Park, Kniebisstraße 67, D-72250 Freudenstadt, Germany; 3 Karl-Liebknecht-Straße 73, D-01109 Dresden. Germany; 4 Steinhardt Museum of Natural History, Tel Aviv University, Ramat-Aviv, Tel Aviv, IL-69978, Israel; 5 Biocentre Grindel, Universität Hamburg, Martin-Luther-King-Platz 3, D-20146 Hamburg, Germany; 6 Department of Biology and Biotechnology, American University of Madaba, P.O.Box 2882, Amman, JO-11821, Jordan; 7 Remez St. 49, IL-36044 Qiryat Tiv’on, Israel; 8 Deichstr. 13, D-21354 Bleckede, Germany

**Keywords:** Middle East, identification key, Geadephaga, species traits, life history traits, application for smartphones and tablets, Android, mobile devices, species status, sibling species

## Abstract

The tiger beetles of the southern Levant (Egypt: Sinai, Israel, Jordan) and adjacent regions of the neighboring countries Lebanon, Syria, Iraq, Saudi Arabia and Egypt are reviewed in terms of species taxonomy, ecological and distributional traits and conservation biology. An illustrated dichotomous identification key from the species of this region is provided. Based on the classical identification key, we developed a digital identification application for smartphones and tablets. The species status of *Calomera aulicoides* (J.R. Sahlberg, 1913) is (re-) established (stat. rest.) as this taxon can be found sympatrically and parapatrically together with *Calomera littoralis winkleri* (Mandl, 1934). Morphological character states are discussed to identify *Cicindela javetii* Chaudoir, 1861 and *C. herbacea* Klug, 1832. *Calomera aphrodisia* (Baudi di Selve, 1864) is recorded for the first time from Israel. The presence of *Calomera aulica* (Dejean, 1831) and *Grammognatha euphratica* (Dejean, 1822) is confirmed by new records. At least five taxa are threatened or extinct in Israel. For one of these species, Israel has a national responsibility for the conservation as the main part of the distribution range is within this country.

Availability: The application TIGER BEETLE ID for Android devices can be freely downloaded at https://doi.org/10.3897/zookeys.734.21989.suppl1. See also disclaimer of warranties.

## Introduction

As tiger beetles are often colorful and diurnal, they have attracted the attention of academics, citizen scientists, and nature-lovers. For example, there is an entire journal ‘Cicindela’ devoted exclusively to this group, and highlights the public interest in these animals which belong to the best known insects (Pearson and Vogler 2001). As several tiger beetles are known to be in strong decline, they became one of the most suitable insect groups for conservation biology (e.g. Cassola and Pearson 2000), including action plans for recovery (e.g. U.S. Fish and Wildlife Service 2009; Vogler and DeSalle 1994; Vogler et al. 1993). Numerous species are listed in European Red Lists or in the U.S. Endangered Species Act.

For many regions, updated identification keys, compilations or even field guides for the tiger beetles are available, e.g. for North America, Australia, parts of China, most parts of Europe and Sub-Saharan Africa (Gebert 2006; Golding 2016; Gourvés 2002; Lisa 2002; Pearson et al. 2015; Pearson et al. 2006; Shook and Wu 2007; Werner 1999; 2000). There have been several publications addressing the tiger beetle fauna of the Levant and the surrounding areas (e.g. Abdel-Dayem et al. 2003; Ali 1978; Deuve 2011; 2012; Gebert 2016; Jaskuła and Rewicz 2014; Matalin and Chikatunov 2016; Nussbaum 1987). However, also after the recently published excellent study of the Israeli tiger beetles with an identification key (Matalin and Chikatunov 2016), there are still some open questions and topics:

(1) In the faunistic part of their work Matalin and Chikatunov (2016) addressed mostly older material (until the 1990s) of the Steinhardt National Collection of Natural History at the Tel Aviv University. Further records, especially from the last two decades, are available and must be taken into account. The southern Levant is a poorly studied region in which Geadephaga species may be overlooked (Schuldt et al. 2009). Therefore species from adjacent countries should be incorporated.

(2) An identification key which includes the species of the adjacent countries would be useful. To meet the need of many enthusiasts and laypersons, for example in Facebook groups which discuss entomology, we present a field guide for mobile devices, such as smartphones and tablets (cf. Farnsworth et al. 2013). Moreover, figures depicting both morphological details and the habitus are provided to bolster the identification skills of the general public.

(3) Open systematic questions, for example, the systematic rank of the two parapatric and sympatric “subspecies” of the *Cicindela littoralis* group in the southern Levant, need to be revised.

(4) Finally, as claimed by both taxonomists and conservationists (e.g. Golding and Timberlake 2003), we incorporate in this taxonomic study comprehensive information about habitat preferences and first assessments to identify threatened species of the tiger beetles of the southern Levant.

## Material and methods

### Delineation of the study area

**Figure 1.**
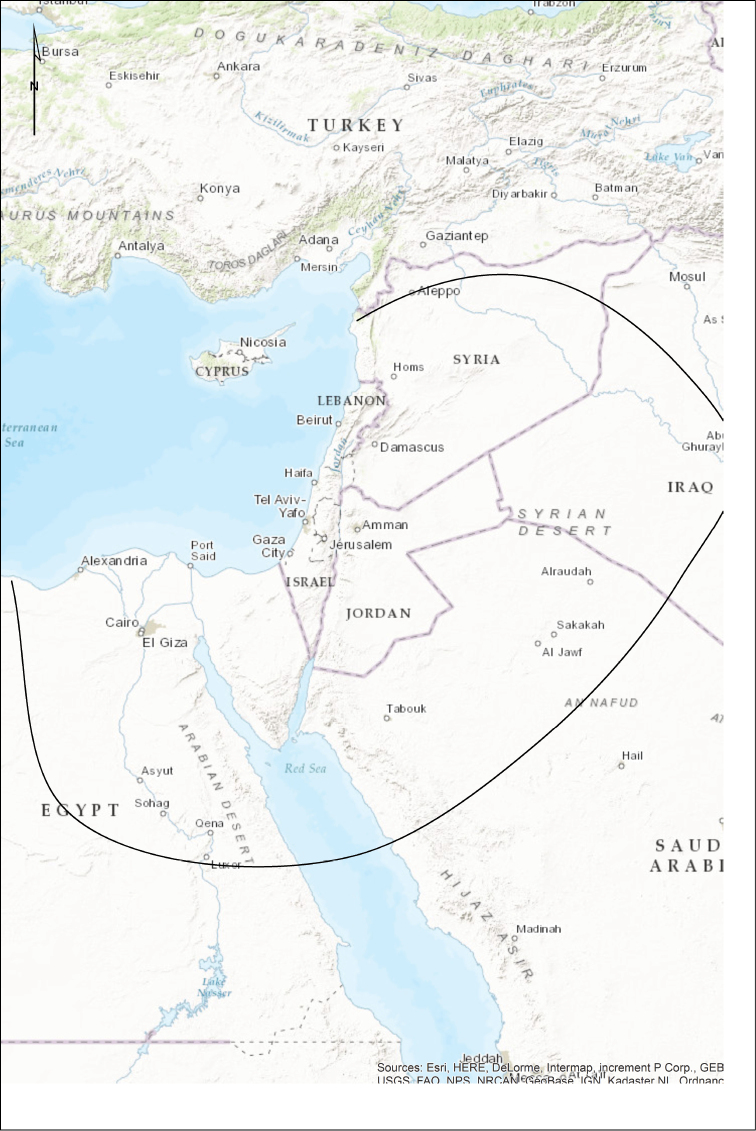
Study area. The line marks the approximate edge of the study area and parts of adjacent lands. Mediterranean islands are excluded.

We define the southern Levant as a section of Southwest Asia comprised of the Sinai Peninsula (Egypt), Israel (including areas under Palestinian control), and Jordan. Species known from surrounding regions in Egypt, Lebanon, Syria, Iraq and Saudi-Arabia are also considered. For an overview of the study area see Fig. 1.

### Nomenclature

We rank the tiger beetles as a family within the Geadephaga. In many other systematic compilations of Adephaga, tiger beetles are ranked as a subfamily of Carabidae (e.g. Ball and Bousquet 2001; Beutel et al. 2007; Lawrence and Slipinski 2013; Müller-Motzfeld 2006). However, recent molecular findings reveal the Cicindelidae, together with Trachypachidae, as the sister taxa to all other Geadephaga (Bocak et al. 2014; López-López and Vogler 2017).

Since Rivalier’s (1950) basic work on male genitalia, the “former” genus *Cicindela* has been split up into small entities, mainly due to differences in the male genitalia (comparable to those within the genus *Carabus*). However, the taxonomy of these genus group names is not consistently used in the literature, especially experts from the New World and many professional biologists still adopt the broad and conservative definition of the genus *Cicindela* (cf. Lorenz 2005; Pearson et al. 2006; Pearson and Vogler 2001; Rivalier 1971; Werner 1991; 1992). We do not want to support this taxonomic “arbitrariness” and try to avoid any superfluous nomenclatural changes. Therefore we adopt the genus (and subgenus) nomenclature from the recent publications of Putchkov and Matalin (2017), Lorenz (2005), and a monography on the Palaearctic tiger beetles in preparation (Gebert, Wiesner, Matalin and Franzen, pers. comm.).

Moreover there are differences in the rank of species and subspecies between many authors (Deuve 2011; Mandl 1981b; Putchkov and Matalin 2003, 2017). If there is evidence for a lack of gene flow in parapatric or sympatric situation we rank the given taxa as species following broadly accepted species concepts (e.g. Biological Species Concept).

### Studied material

This study is based on the examination of specimens collected during the authors’ field trips in Egypt, Israel, and Jordan, as well as specimens stored in entomological collections (including material from Europe, Africa, and other parts of Asia for comparisons). We studied approximately 2,000 specimens stored in the following collections:

**CAL** Working collection Assmann, Lüneburg, Germany (part of ZSM)

**CGD** Working collection Gebert, Dresden, Germany

**COQ** Working collection Orbach, Qiryat Tiv’on, Israel (will be transferred to SMNHTAU, Israel)

**CSW** Working collection Starke, Warendorf, Germany (will be transferred to Westfälisches Landesmuseum Münster, Germany)

**CSH** Working collection Schnitter, Halle/S., Germany

**CWB** Working collection Wrase, Berlin, Germany (part of ZSM)

**SMNHTAU** Steinhard Museum of Natural History, National Collections, Tel Aviv University, Tel Aviv, Israel

**NHMP** Muséum National d’Histoire Naturelle, Entomology Department, Paris, France

**ZISP** Zoological Institute of the Academy of Sciences, St. Petersburg, Russia

**ZSM** Zoological State Collection Munich (Zoologische Staatssammlung München), München, Germany

We received information from colleagues about few records from the following collections:

**SDEI** Senckenberg German Entomological Institute (Deutsches Entomologisches Institut), Müncheberg, Germany

**ZISP** Coleoptera Department, Laboratory of Insect Taxonomy, Zoological Institute of the Russian Academy of Sciences, St. Petersburg, Russia

### Measurements, photographs, distribution records, traits

For detailed explanations about measurements, photography, traits and distributional data see other publications about the carabid beetles of the southern Levant (Assmann et al. 2012; 2015a; 2015b).

### Criteria to classify threatened species

As few biologists and citizen scientists work on tiger beetles in the Middle East, our data do not allow for the estimation of a trend for all species. Thus, we used the approach of Ludwig et al. (2006) to classify threatened species for Red Lists. This approach is based on recent abundance, short-term and long-term trends of populations and habitats as well as the risk factors for the given species. If long-term data (50–150 years) are not available, we set this criterion to ‘data deficient’ for the identification of the threat categories. Sufficient data on the threat to cicindelid beetles are only available from Israel. Therefore, the assessments are made only for Israel.

If large parts of the distribution range (>67 %) of a taxon are in the study region we derive the national responsibility of the given states for the preservation of the given taxa (cf. Gruttke et al. 2004; Schnittler and Günther 1999).

### Identification keys

We developed two tools for the identification of the tiger beetles in the southern Levant:

(a) A “classical”, dichotomous identification key (Winston 1999) containing textual descriptions and figures. We used simple terminology of morphological characters and their states to make the key user-friendly.

(b) An Android application for mobile phones and tablets (Android Studio environment: Google and Alliance 2016, Android Homepage). Text and figures are adopted from the “classical” identification key.

## Results

### Characterization of the Cicindelidae species in the southern Levant

**Figures 2, 3.**
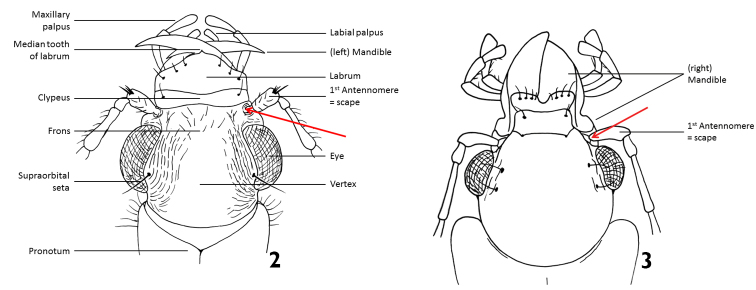
Head of a tiger beetle (left) and of a ground beetle (right) (dorsal view = upper side) and structures often used for identification. The arrows mark the insertion of antenna.

In general, tiger beetles differ from all other ground beetles in the position of antennae which insert on the frons of head, between the bases of mandibles (Fig. 2). In other ground beetles, the antennae insert in line with and posteriad adjacent mandibular bases (Fig. 3).

Tiger beetles are agile, usually diurnal beetles with a head (including the eyes) wider than the pronotum; long, thin legs and long, sickle-shaped mandibles with long, simple teeth along the inner edge of the mandibles (Figs 2, 3). All species from the Middle East have fully developed wings which are used during short flights for hunting and escape flights.

The habitus photographs (Figs 7–38) provide further assistance in the identification of cicindelids. An overview of the external morphology of cicindelids and the relevant terminology is found in Figs 2 to 5.

**Figure 4.**
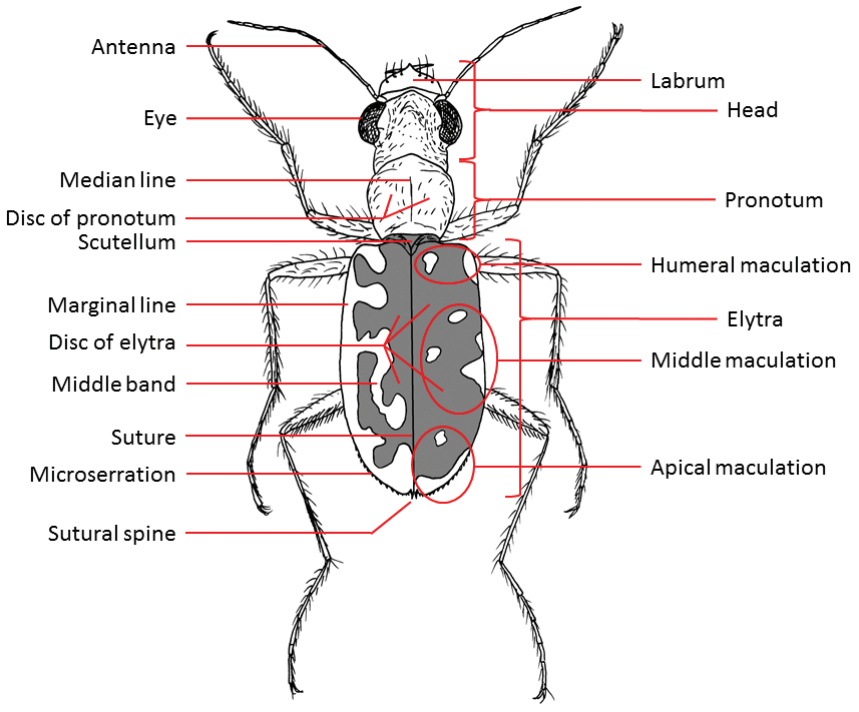
Habitus of a tiger beetle (dorsal view = upper side) and structures often used for identification.

**Figure 5.**
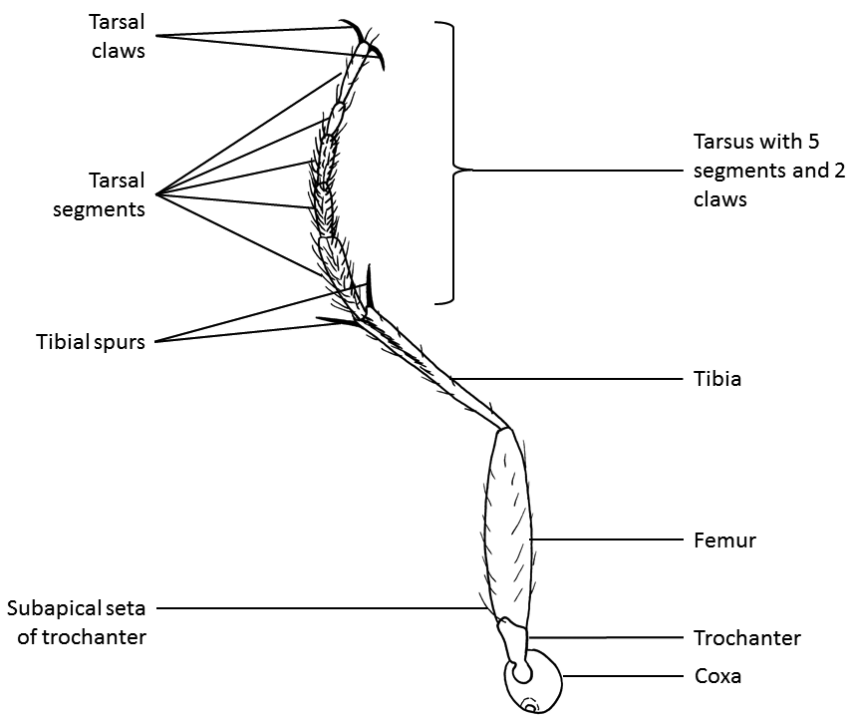
Leg of a tiger beetle and structures often used for identification. The prefixes pro-, meso- and meta- are used to indicate parts of the front, middle and hind legs, respectively. For example, metatibia refers to the tibia of hind leg and profemur to the femur of fore leg.

**Figure 6.**
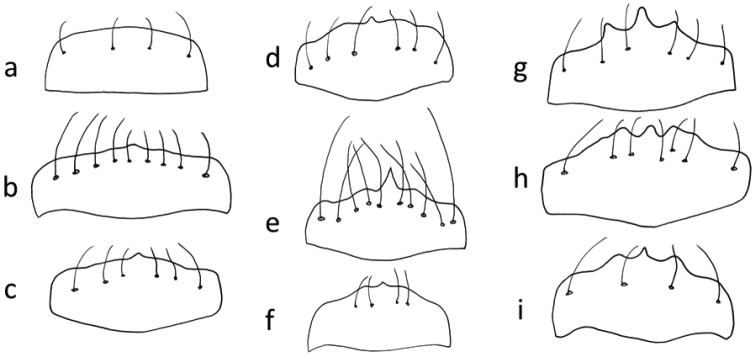
Labrum of tiger beetles: **a** without a tooth (*Myriochila melancholica*) **b, c, d, e, f** with 1 tooth (**b** *Cylindera contorta valdenbergi* **c** *Cephalota littorea* **d** *Cicindela javetii* **e** *Cylindera rectangularis* **f** *Cephalota vartianorum*) **g, i** with 3 teeth (**g** *Cicindela asiatica* **i** *Myriochila orientalis*) **h** with 3 to 5 teeth (**h** *Cephalota tibialis*).

### Identification key to the tiger beetles from the southern Levant and adjacent territories

For ease of orientation, the numbering schemes of the species found in the identification key and in the species accounts are identical. Species which are known from adjacent countries, but not from the southern Levant itself are given in parentheses.

Chikatunov et al. (2006) and Ptashkovsky (2013) indicated 29. *Cephalota deserticola* (Faldermann, 1836) for Israel. However, its distribution range stretches from western Iran to Central Asia and China (Gebert 2016; Werner 1992; Wiesner 1992) and therefore, based on geographical considerations, we consider it is unlikely that the species is found in Israel. As no verifiable records from the southern Levant have been preserved in SMNHTAU (including the recently transferred collection of Ptashkovsky), we treat the published records for *C. deserticola* as misidentifications (cf. Matalin and Chikatunov 2016), and do not include this species in the identification keys. Moreover, in SMNHTAU there are no *Cephalota deserticola* specimens with an identification label from Mandl (own observation).

The following species are also not incorporated in the key: 30. *Cylindera pygmaea* (Dejean, 1825), 31. *Calomera caucasica* (Adams, 1817), the *Salpingophora* species 32. *S. bellana* (W. Horn, 1905), 33. *S. hanseatica* (W. Horn, 1927) and 34. *S. rueppelii* (Guérin-Méneville, 1847), and the *Hypaetha* species 35. *H. schmidti* (W. Horn, 1927) and 36. *H. copulata* (Schmidt-Göbel, 1846). These species are recorded from the adjacent countries (e.g. Putchkov and Matalin 2003, 2017; Wiesner 1992), but exclusively from their distant parts (e.g. Caucasus Mountains, the south-eastern coast of the Arabian Peninsula), and thus their occurrence in the southern Levant is unlikely. In many cases, the photographs from Werner (1991; 1992) may be sufficient to identify these species.

**Figures 7–10.**
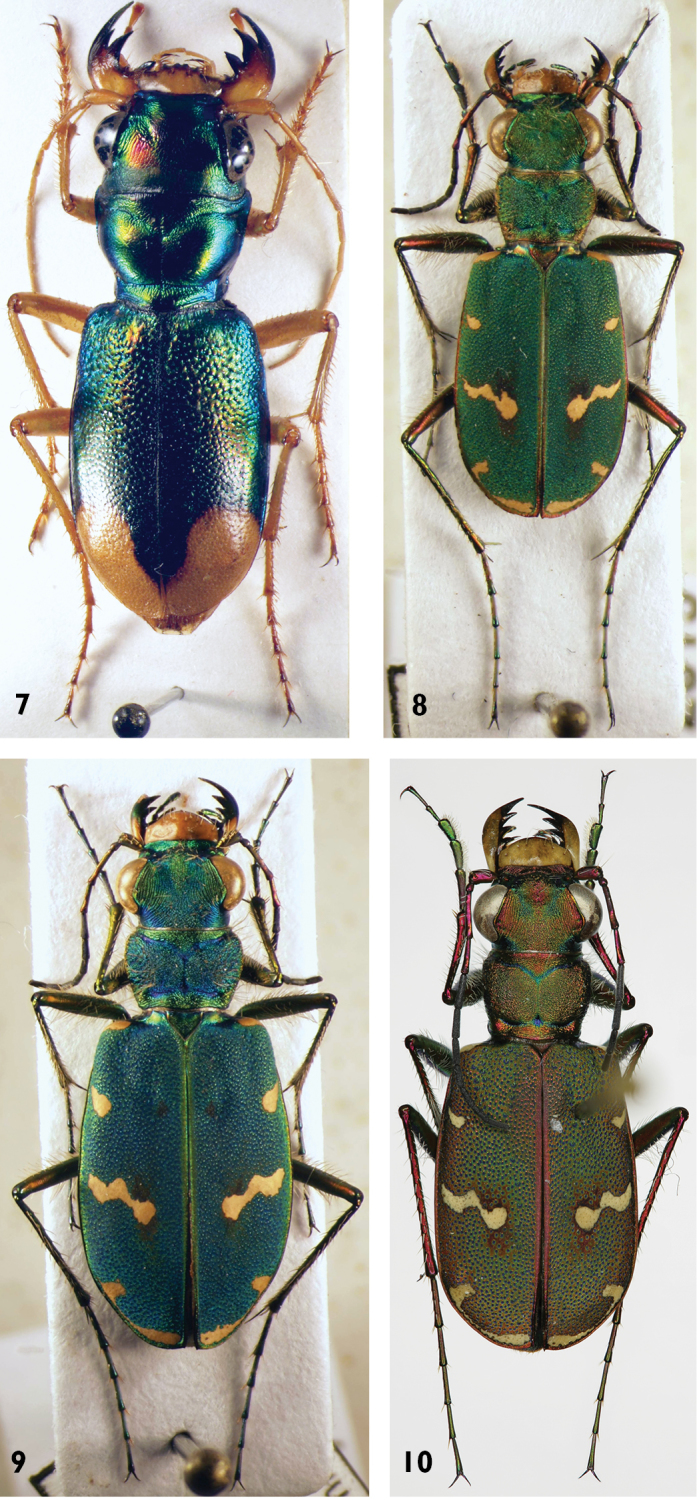
Cicindelidae species: **7** *Grammognatha euphratica* (female) **8** *Cicindela javetii* (male) **9** *C. javetii* (female) **10** *C. javetii* (male, paratype of *thughurica*).

**Figures 11–12.**
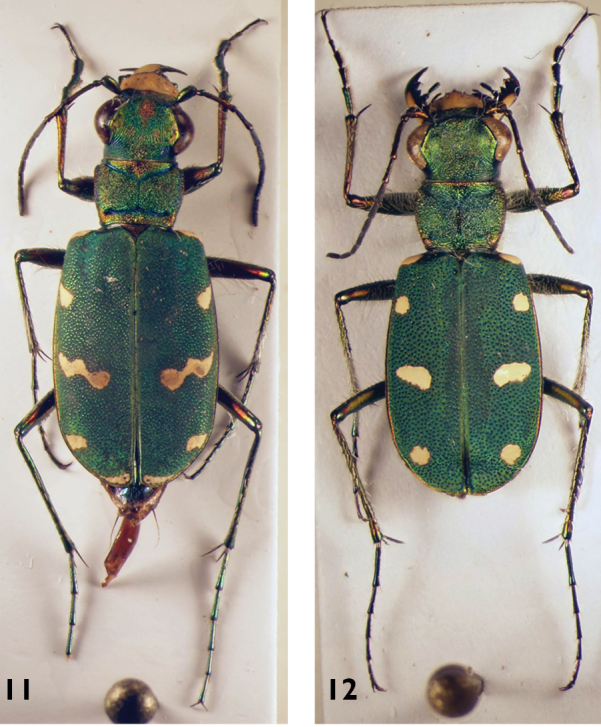
*Cicindela* species: **11** *C. herbacea* (male) **12** *C. asiatica* (male).

**Figures 13–16.**
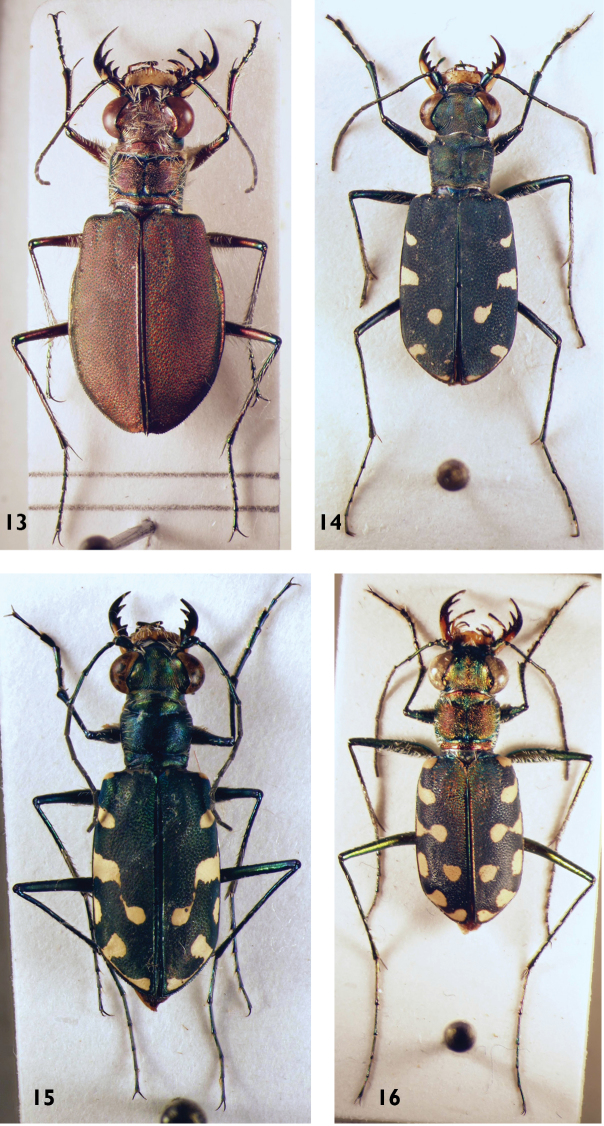
*Calomera* species: **13** *C. concolor* (female) **14** *C. fischeri* (male) **15** *C. alboguttata* (male) **16** *C. aulica* (male).

**Figures 17–20.**
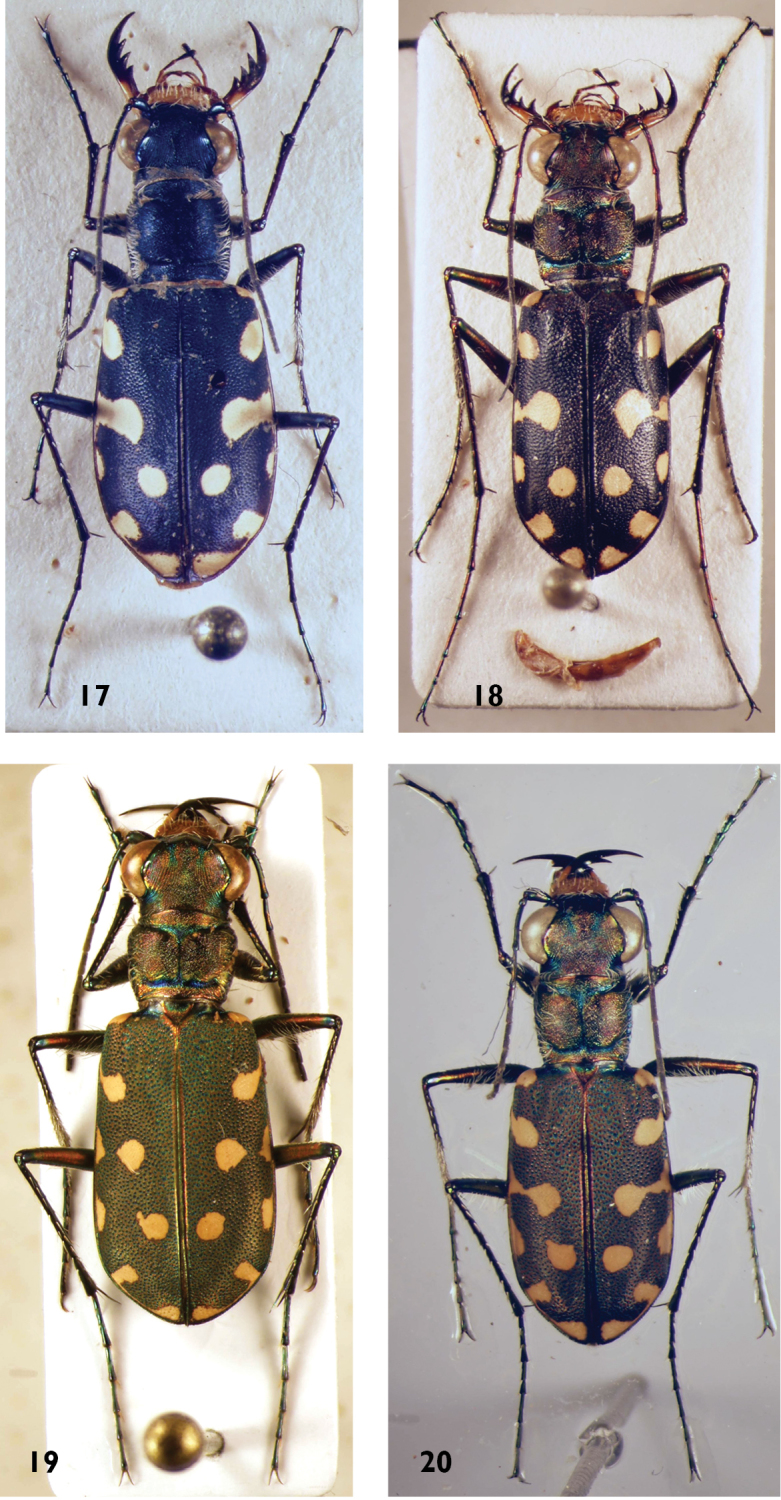
*Calomera* species: **17** *C. diania* (male) **18** *C. aphrodisia* (male) **19** *C. littoralis winkleri* (male) **20** *C. aulicoides* (male).

**Figures 21–24.** Cicindelid species: **21** *Calomera fimbriata* (male) **22** *Habrodera nilotica* (female) **23** *Homodela ismenia* (male) **24** *Hypaetha singularis* (female).

**Figures 25–28.**
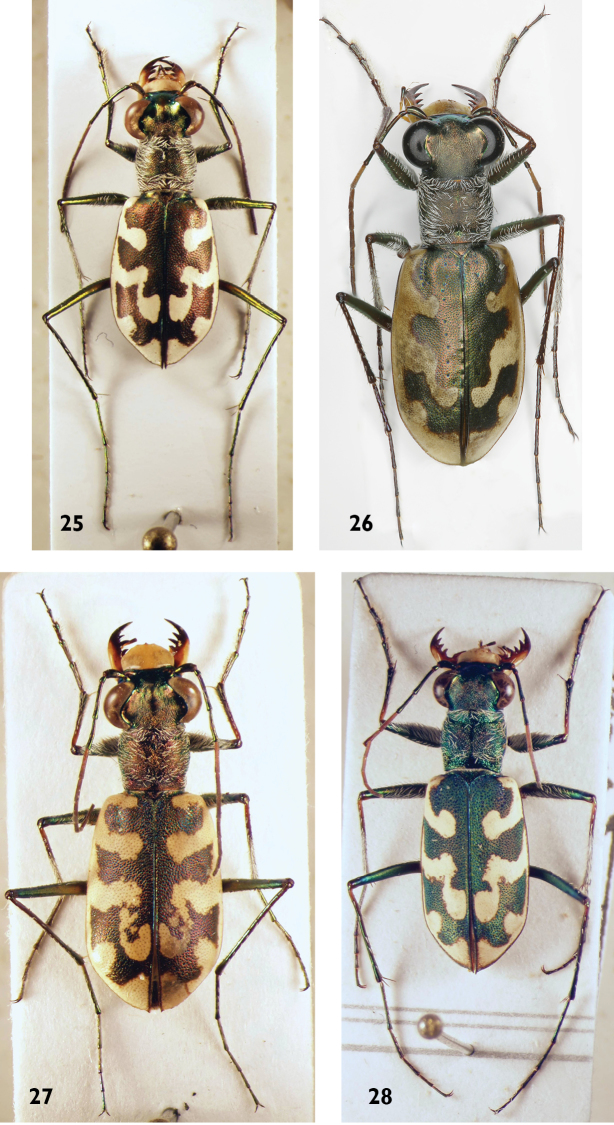
*Cephalota* species: **25** *C. littorea* (male) **26** *C. tibialis* (male) **27** *C. circumdata* (male) **28** *C. vartianorum* (male).

**Figures 29–32.**
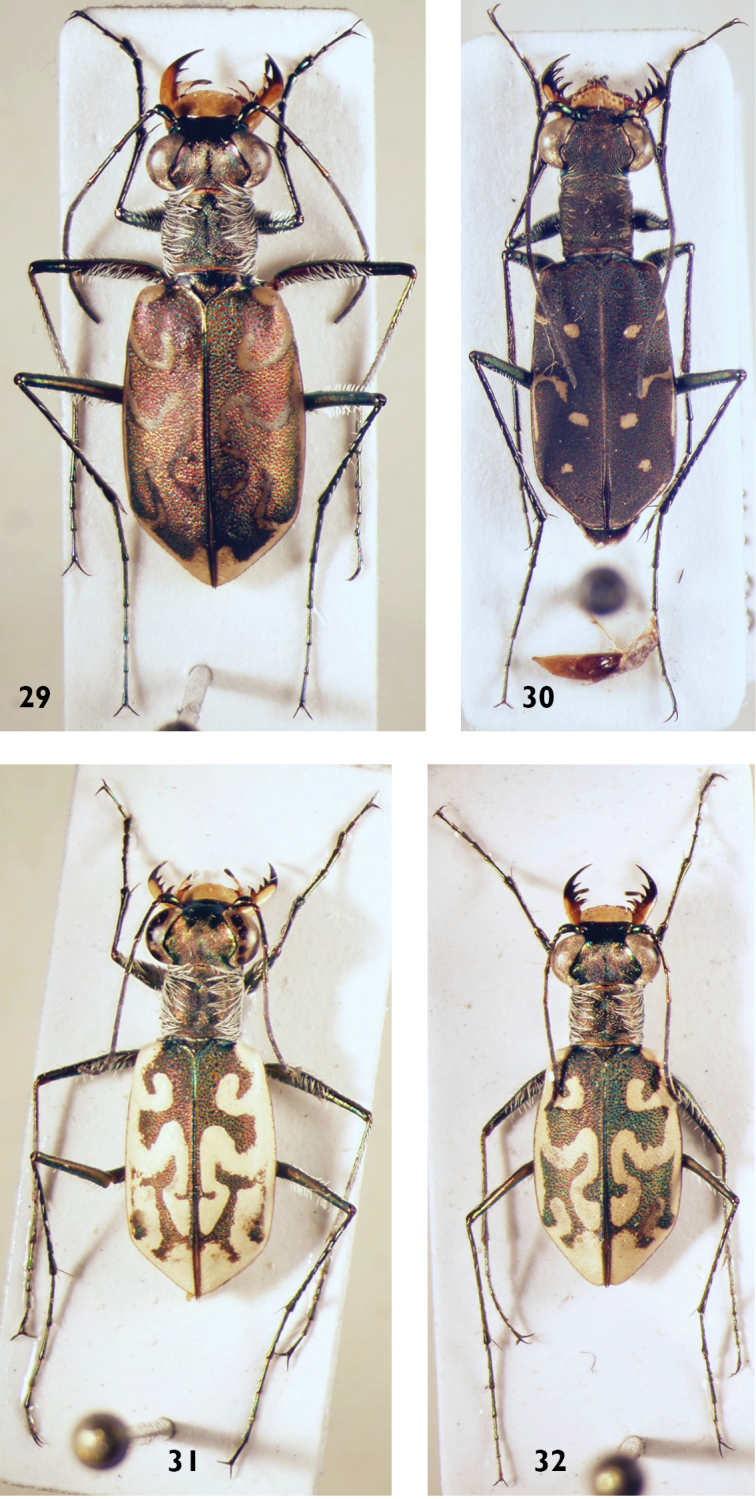
*Cylindera* (sub-) species: **29** *C. contorta* *s.str.* (male) **30** *C. rectangularis* (female) **31** *C. contorta valdenbergi* (male) **32** *C. contorta valdenbergi* (female).

**Figures 33–35.**
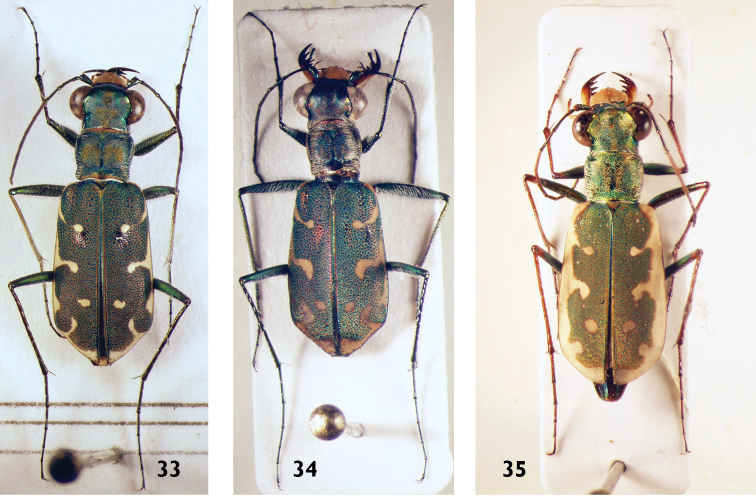
*Myriochila* species: **33** *M. melancholica* (female) **34** *M. orientalis* (female) **35** *M. dorsata* (female).

**Figures 36–38.**
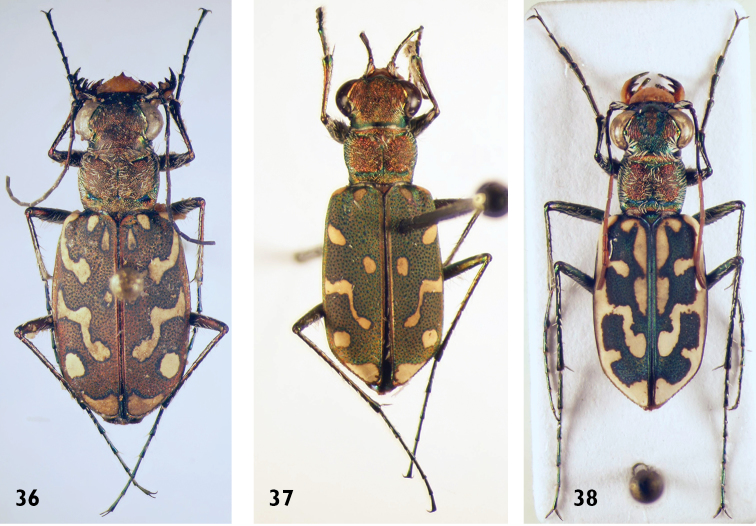
*Lophyra* species: **36** *L. flexuosa* (female) **37** *L. hilariola* (male) **38** *L. histrio* (male).

**Table:** 

1	Larger (19–26 mm). Scutellum not visible in commonly mounted beetles, not triangular. Side margin of pronotum with a forward projecting lobe. Last segment of maxillary palpi shorter than penultimate one. Elytra green (rarely blue or black) with a pale apical spot. Fig. 7	**1. *Grammognatha euphratica* (Dejean, 1822)**
–	Smaller (less than 18 mm). Scutellum clearly visible and triangular. Side margin of pronotum weakly developed, without a forward projecting lobe. Penultimate segment of maxillary palpi as long as the last one or shorter. Coloration different, if green then with more than 1 pale spot	**2**
2	Pronotal margin clearly visible on upper side of prothorax, its lateral sides (hypomeron/epimeron) visible from above; anterior margin of pronotum with a dense and regular series of white setae (Fig. 55). Pale pattern of elytra is expanded, on the disc middle band connected with the apical band, but along the suture regularly dark. 7.5–10 mm. Figs 24, 47	**25. *Hypaetha singularis* (Chaudoir, 1876)**
–	Pronotal margin sometimes difficult to detect, but its lateral sides (hypomeron/epimeron) not visible from above; anterior margin of pronotum without white setae or, if they are present, they are irregularly positioned. Middle and apical bands of elytra never broadly connected	**3**
3	Elytra with pale border along the outer edge, not interrupted by dark sections	**4**
–	Elytra with or without pale border along the outer edge, if present then interrupted by dark sections	**11**
4	Frons (area of forehead between the eyes) with white setae close to the fore margin of the eyes and close to the hind margin (Fig. 55). 1^st^ antennal segment with numerous setae. Genae (lateral side beneath the eyes) with dense white setae (Fig. 48). 7.5–8.5 mm. Fig. 22	**14. *Habrodera nilotica* (Dejean, 1825)**
–	Frons without white setae. 1^st^ antennal segment with one or several erect distal setae. Genae without or few setae	**5**
5	1^st^ antennal segment with several white setae and the usual erect distal seta (sometimes they are broken, but their insertions are still visible) (Fig. 54)	**6**
–	1^st^ antennal segment with only one erect distal seta (Fig. 54)	**7**
6	Frons (area of forehead between the eyes) glabrous, also along the hind margin of eyes glabrous, only with supraorbital setae. Labrum with 1 tooth (cf. Fig. 6). Elytral pale pattern regularly without spots, only bands. 12–15 mm. Figs 27, 43c	**17. Cephalota (Taenidia) circumdata (Dejean, 1822)**
–	Frons with white setae at the hind margin of eyes, in addition to the supraorbital setae. Labrum with 3 teeth. Elytral pale pattern with at least one pair of discal spots. 10–13 mm. Figs 38, 45c	**28. *Lophyra histrio* (Tschitschérine, 1903)**
7	Elytral pale pattern on the disc reduced and constricted, forming spots which are (partly) connected with the pale margin. 9–13 mm. Fig. 35	**24. Myriochila (Monelica) dorsata (Brullé, 1834)**
–	Elytral pale patterns not or slightly constricted, forming complex bands, not spots; at least middle band bent downwards, sometimes s-shaped (Figs 25, 26, 28, 31, 32)	**8**
8	Labrum without or with 1 median tooth (Fig. 6b, c, f)	**9**
–	Labrum with 3 or more teeth (Fig. 6h). Pale elytral margin wide. Longitudinal row of punctuation parallel to elytral suture. 11–15 mm. Figs 26, 43b	**16. Cephalota (Taenidia) tibialis (Dejean, 1822)**
9	Labrum with more than 8 setae (Fig. 6b). Elytra in the apical third angularly pointed. Pale elytral pattern strongly bent, middle band s-shaped. 9–10.5 mm. Figs 6b, 31, 32, 44a	**20. Cylindera (Eugrapha) contorta (Fischer von Waldheim, 1828), ssp. valdenbergi (Mandl, 1981)**
–	Labrum with less than 8 setae (Fig. 6c, f). Elytra more evenly rounded. Pale pattern with only slightly bent bands. Specimens regularly larger than 10 mm	**10**
10	Maximum width of head (across the eyes) more than 1.3 times wider than pronotum. Fore margin of labrum weakly curved. Apical tooth of the elytra sharply pointed. Elytral pale marginal pattern behind the basal band wider. Antennomeres 5 and following ones less contrasting in coloration from the first four ones. Surface shinier. 10–12 mm. Figs 6c, 25, 43a	**15. Cephalota (Taenidia) littorea (Forskål, 1775)**
–	Maximum width of head (across the eyes) less than 1.3 times wider than pronotum. Foremargin of labrum strongly curved. Apical tooth of the elytra evenly pointed. Elytral pale marginal pattern narrower behind the apical band strongly constricted, sometimes interrupted. Antennomere 5 contrasting different in coloration from the first four ones. Surface less shiny (dull). 10–14 mm. Figs 6f, 28, 43d	**18. Cephalota (Taenidia) vartianorum (Mandl, 1967)**
11	Elytra red to brown or greenish, without any pale pattern (neither bands nor spots). White setae from clypeus to hind margin of eyes. 10–14.5 mm. Fig. 13	**5. *Calomera concolor* (Dejean, 1822)**
–	Elytra with pale spots, bands or complex patterns. Sometimes white setae on frons	**12**
12	Genae (lateral side beneath the eyes) with some white setae (Fig. 48)	**13**
–	Genae without distinct setae (sometimes with single setae)	**20**
13	White setae between clypeus and eyes and around the antennal basis (Fig. 55)	**14**
–	White setae on labrum and clypeus, but not between clypeus and eyes or if so, than not around the antennal basis (Figs 50, 51)	**16**
14	Large species: 15–18 mm. Fig. 21	**13. *Calomera fimbriata* (Dejean, 1831)**
–	Smaller species: <15 mm	**15**
15	Apical margin of labrum with a median tooth which is rarely reduced. Head 1.3–1.4 times wider than pronotum. 6 spots at or close to the elytral margin, a marginal spot at the level of the discal spot. In general, two pairs of elytral spots connected with each other: the apical spots as well as the discal and 3^rd^ marginal spots (counted from the base towards the apex), but sometimes the extensions between the given spots interrupted. Larger: 11–15 mm. Figs 15, 55	**7. *Calomera alboguttata* (Klug, 1832)**
–	Apical margin of labrum smooth. Head less than 1.3 times wider than pronotum. 5 pale spots on the elytral margin. All pale elytral spots isolated from each other, only the apical spots sometimes with an (interrupted) extension. Smaller: 8–12 mm. Fig. 14	**6. *Calomera fischeri* (Adams, 1817)**
16	Posterior part of the metafemur with one complete series of shorter white setae; some setae belonging to a second parallel series occur mainly at the base, but this series is not complete (Fig. 49 left)	**17**
–	Posterior part of metafemur with two almost complete parallel series of longer white setae (Fig. 49 right)	**19**
17	Labrum with less than 25 setae (Figs 50 right, 51). Elytra rounded or parallel-sided. Pronotum shorter (width of pronotum/length of pronotum PW/PL: >1.05). Elytral shoulders less prominent. Elytra dark, not bluish. Median lobe of aedeagus slender and stretched (Fig. 40a, c)	**18**
–	Labrum with more than 26 setae (Fig. 50 left). Elytra more enlarged in the apical half (dorsal view) and convex (lateral view), pronotum sides less rounded and longer (PW/PL: <1.05). Elytral shoulders prominent. Elytra and forebody bluish. Median lobe of aedeagus wider in the middle and more rounded (Fig. 40b). 12–13.5 mm. Fig. 17	**9. *Calomera diania* (Tschitschérine, 1903)**
18	Elytra less elongate and more ovate. Pronotum more transverse with more rounded sides, body flatter. Forebody more colourful, often with green and red lustre. Width of head/width of pronotum ratio >1.19. Median lobe of aedeagus less rounded (Fig. 40a). 11–13.5 mm. Fig. 16	**8. *Calomera aulica* (Dejean, 1831)**
–	Elytra more elongate and less ovate. Pronotum more parallel-sided. Forebody and elytra darker. Width of head/width of pronotum ratio <1.18. Median lobe of aedeagus more rounded (Fig. 40c). 14–16 mm. Figs 18, 51	**10. *Calomera aphrodisia* (Baudi di Selve, 1864)**
19	Long metatibial spur longer, about 2/3 of length of 1^st^ metatarsal segment (hind legs, Fig. 53). 3 teeth on inner side of left mandible, rarely a small fourth tooth developed (Fig. 52). In general, elytral pale spots more isolated; the two discal spots isolated from each other and marginal spots, the extension between the two median marginal spots normally interrupted. Median lobe of aedeagus similar to that one of *C. aulicoides*, but with apical part more strongly bent (Fig. 41). Copulatory piece of median lobe of aedeagus with 2 (or 3) tips which are sideward oriented (Fig. 42 below). 10–13 mm. Fig. 19	**11. Calomera littoralis (Fabricius, 1787), ssp. winkleri (Mandl, 1934)**
–	Long metatibial spur shorter, about half of the length of 1^st^ metatarsal segment (hind legs, Fig. 53). 4 teeth on inner side of left mandible (Fig. 51). In general, elytral pale spots more connected: the fore discal spot with an extension to the neighboring marginal spot; the marginal spots connected to three pairs (humeral, medial, and apical lunules). Median lobe of aedeagus similar to that one of *C. littoralis*, but with apical part less bent ventrally (Fig. 41). Copulatory piece of median lobe of aedeagus straight or slightly curved, tip broadly rounded (Fig. 42 top). 9–13 mm. Fig. 20	**12. *Calomera aulicoides* (J.R. Sahlberg, 1913), stat. rest.**
20	Elytra green with pale pattern reduced to spots or small bands and small reddish areas (Figs 8–11, 23)	**21**
–	Elytral coloration different, not green, if so then pale patterns larger and complex	**24**
21	Labrum with 3 teeth on anterior margin (Fig. 6). Each elytron with 2 to 4 pale spots which are not connected	**22**
–	Labrum with one tooth on anterior margin (Fig. 6). Each elytron with (4 to) 5 pale spots, the 2 apical spots usually connected at the external margin	**23**
22	Each elytron with 2 (to 3) pale spots. Frons without setae. 1^st^ antennal segment with one erect distal seta. 9.2–13.5 mm. Fig. 23	**19. *Homodela ismenia* (Gory, 1883)**
–	Each elytron with 4 pale spots. Frons with few setae. 1^st^ antennal segment with few setae. 14–18 mm. Figs 6g, 12	**4. *Cicindela asiatica* Audouin & Brullé, 1839**
23	Pronotum more cordiform, its sides more convex, fore margin (apically to the protruding fore angles) of similar width as (or a little bit wider than) posterior margin. Head in relation to pronotum wider than in *C. herbaceae*. Internal sac of median lobe of aedeagus shorter (lateral view), median lobe less than 3 times longer than structures of internal sac (not evaginated), shape of median lobe in lateral view more rounded, the apex sharper and stronger downward bent (Fig. 39a, b). 11–15 mm. Figs 6d, 8–10	**2. *Cicindela javetii* Chaudoir, 1861**
–	Pronotum less cordiform, its sides less convex, fore margin (apically to the protruding fore angles) wider than hind margin (or, rarely of about the same width). Head in relation to pronotum less wide than in *C. javetii*. Internal sac of median lobe of aedeagus longer, median lobe more than 3 times longer than structures of internal sac (not evaginated), shape of median lobe more stretched and slender, the apex more rounded and less downward bent (Fig. 39c). 13.5–17 mm. Fig. 11	**3. *Cicindela herbacea* Klug, 1832**
24	1^st^ antennal segment with several white setae (Fig. 54 below)	25
–	1^st^ antennal segment with 1 distal seta only (Fig. 54 above)	26
25	Approximately 5 to 15 white frontal setae at hind margin of eyes. 1^st^ antennal segment with numerous setae. 11–14 mm. Figs 36, 45a	**26. *Lophyra flexuosa* (Fabricius, 1787)**
–	Approximately 2 to 4 white frontal setae at hind margin of eyes. 1^st^ antennal segment with few setae. 10–12 mm. Figs 37, 45b	**27. *Lophyra hilariola* (Bates, 1874)**
26	Pale elytral margin only along a short section of the basal half interrupted. (See also no. 10 of the key). 10–14 mm. Figs 6f, 28, 43d	**18. Cephalota (Taenidia) vartianorum (Mandl, 1967)**
–	Pale elytral margin along two sections interrupted, both along the basal and the apical part	27
27	Labrum with (6-) 8 - 10 (-12) long hairs, both sexes with one tooth (Fig. 6e). Pale pattern on elytra strongly reduced. Elytral coloration dark brown. 7–10 mm. Figs 6e, 30, 44b	**21. Cylindera (Ifasina) rectangularis (Klug, 1832)**
–	Labrum with (2-) 4 hairs, females with 3 teeth and males with 1 tooth or without teeth (Fig. 6a,i). 9–13.5 mm	**28**
28	Pale pattern on elytra narrower; basal pale spot of elytra often separated from the humeral lunule; middle band often interrupted and forming both a discal spot and a short maculation; females on the basal third of elytral disc with a smaller smooth, polished shiny area. Elytra towards the apical part less enlarged in both sexes. Smaller: 9–12.5 mm. Figs 6a, 33, 46a	**22. Myriochila (s.str.) melancholica (Fabricius, 1798)**
–	Pale pattern on elytra wider, basal pale spot of elytra often linked to the humeral lunule; middle maculation slightly constricted, only rarely interrupted; females on the basal third of elytral disc with a wider smooth, polished shiny area. Elytra towards the apical part in both sexes stronger enlarged. Larger: 10–13.5 mm. Figs 6i, 34, 46b	**23. Myriochila (Monelica) orientalis (Dejean, 1825)**

### TIGER BEETLES ID: the application for smartphones and tablets

The above presented key for the tiger beetles of the southern Levant and adjacent territories is also available as a stand-alone application (app) for portable Android devices (Android-version 5.0 and later releases; Application Programming Interface (API) of 21 or higher is recommended), and can be downloaded from https://doi.org/10.3897/zookeys.734.21989.suppl1. On most devices, the app requires less than 150 Mega bytes (MB) of storage.

After the loading screen, the users will first see a short morphological definition of cicindelids and drawings of the external morphology with key terms indicated (see above). The next screen leads to the dichotomous identification key and to the species list. All photographs and most of the drawings have a zoom function which enables viewing at a higher resolution. Each species name is linked to the species’ accounts with information about habitat, distribution and conservation status (shortened version of the species accounts given below). Here too, a habitus photograph which can be enlarged allows for better orientation and helps to verify identification to species level. The species list is probably be more helpful for experienced users, while beginners should start with the identification key. Figure 56 contains screenshots from the app, giving an overview of its architecture.

### Species accounts

All species are macropterous and flight active. If the species are not recorded from the southern Levant, or if not enough data about the populations during the last decades are available, no conservation information is given.

#### 1. Grammognatha euphratica

Taxon classificationAnimaliaColeopteraCicindelidae

(Dejean, 1822)

##### Habitat.

In salty habitats, on the Mediterranean coast in marshlands (often with *Anthrocnemum*). Around the Dead Sea and in the Arava Valley in salty wetlands and in date palm plantations (own observations). Nocturnal. Attracted by light.

##### Phenology.

Teneral individuals in early spring (Cyprus: February), adults are active until approximately November (own observations). The number of eggs laid per females in a laboratory experiment ranges from 3 to 25 (Aydın 2011a), which is relatively low for an insect.

##### Distribution range.

From southern Spain, Morocco and Sardinia to Central Asia (Cassola 1981; Cassola et al. 2014; Franzen 2001b; Franzen and Gigli 2003; Putchkov and Matalin 2003, 2017).

##### Distribution in the southern Levant.

Mediterranean Sea coast of the Sinai Peninsula (eastwards to El-Arish) and close to Haifa (Atlit); Red Sea coast of the Sinai Peninsula (incl. near Eilat); in the Dead Sea area (especially in the swamps south of the Dead Sea) and in the Arava Valley (Franzen 2001b; Nussbaum 1987). Putchkov and Matalin (2003, 2017) list the species for Jordan. Nasir and Katbeh-Bader (2017) cited Putchkov and Matalin (2003), but do not know a record from Jordan. We do not know of any verifiable record from this period. Our record for Jordan: “29.03.2016, Pot Ash City environs, Dead Sea (Tamarisk bushes), saltmarshes, close to the edge of the sink holes in mud clefts” (CGD).

##### Taxonomic notes.

*Grammognatha* Motschulsky, 1850 is frequently ranked as a subgenus of *Megacephala* Latreille, 1802, but see Gillett (2009). Darker colored morphs occur frequently in the Dead Sea region and resemble the eastern subspecies armenica (Laporte de Castelnau, 1834) which occurs westwards to Iran. The dark form also occurs on the Mediterranean Sea coast, though it is rare.

##### Conservation.

Endangered in Israel. The species is sensitive to disturbances (drainage of habitats, cattle grazing, etc.) (Aydın 2011b). The populations found along the Mediterranean coast of Israel are in decline, and there is only one known new record in the last two decades (Atlit, late May 2012, record in collection Aligi Bandinelli). Numerous habitats have been destroyed in the Dead Sea region, but the species can sometimes be found in date palm plantations.

#### 2. Cicindela javetii

Taxon classificationAnimaliaColeopteraCicindelidae

Chaudoir, 1861

##### Habitat.

Open habitats with dwarf shrubs and bare ground, mostly on loamy soils (own observations and Chikatunov pers. comm.), also in quarries. Israeli records from about 1000 m a.s.l. upwards, in Lebanon up to about 2200 m a.s.l. Diurnal.

##### Phenology.

Adults found mainly in May (April to June, own observation, Matalin and Chikatunov 2016). No verifiable records for the long activity period reported by Nussbaum (1987).

##### Distribution range.

Southern Turkey, Lebanon, southwestern Syria, and northern Israel (Deuve 2011).

##### Distribution in the southern Levant.

Some records have been published by Deuve (2011). In Israel the species is only known from two areas: Mount Hermon (in the surrounding of Majdal-Shams) and from Mount Meron (Nussbaum 1987).

##### Taxonomic notes.

The *C. campestris* Linné, 1758 group in Asia Minor and the Middle East has been the object of recent studies (e.g. Deuve 2011; Franzen 2007; Matalin and Chikatunov 2016), but the taxonomic status of some populations has not yet been completely resolved. We agree with Azadbakhsh and Nozari (2015) that this entire tiger beetle group from South-west Asia needs to undergo revision. We believe that morphometric and molecular studies are necessary to solve the actual taxonomic and systematic problems of this group. Moreover, large amounts of material are needed for studies as the morphometric variability within populations is large (Franzen 2007).

Following Deuve (2011: 136) the specimens from Israel belong to *C. javetii azari* Deuve, 2011. Two further subspecies occur from Turkey to Lebanon and Syria: *C. j. thughurica* Franzen, 2007 and the nominate subspecies. The taxon *thughurica* (Fig. 10) described from southern Turkey, has been also recorded from south-western Syria (Bludan, north-west of Damascus) (Avgin and Wiesner 2009). The given site is close to the Israeli border. The existence of two subspecies of this flight-active species in this small geographic area seems unlikely. Moreover, the elytral pattern, one of the main characters to distinguish the subspecies, is not constant but varies even within a given population clearly (Figs 8, 9).

**Figure 39.**
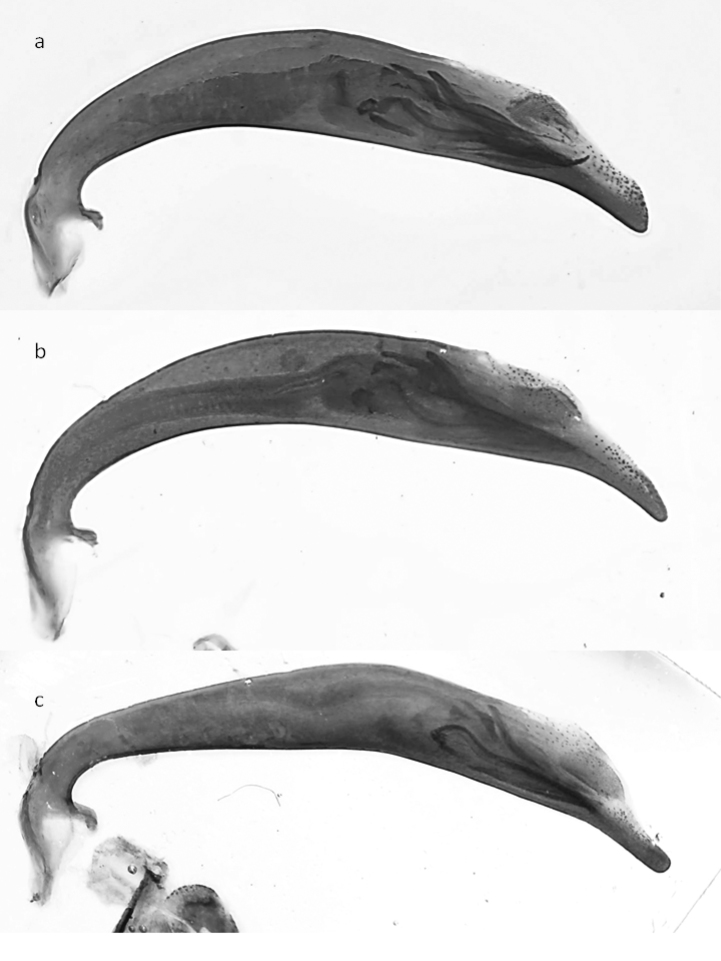
Median lobes of the aedeagus of *Cicindela species*: **a, b** *C. javetii* and **c** *C. herbacea*.

The very similar species *C. herbacea* occurs from Lebanon and Syria to Iran including several populations and described subspecies (Deuve 2011; 2012). The separation of these two species may be possible using male genitalia. Deuve emphasized the size and external shape of median lobe of aedeagus (cf. Figs 24–30 in Deuve 2011). Matalin and Chikatunov (2016) also emphasized the length of the median lobe as a distinguishing character. However, we have specimens from northern Lebanon (Les Cedres, Bcharre) in which the median lobe is shorter than indicated by the latter authors. Also the external shape of the median lobe of both species, *C. herbacea* and *C. javetii*, varies greatly, even within a population (e.g. Bludan, Antilebanon, 1700–2300m, Fig. 39), and not only between the populations of *javetii* (cf. Deuve 2011). However, the median lobe of *javetii* is in general more bent than that of *herbacea*. Although the ventro-lateral bladders of the median lobes (Matalin and Chikatunov 2016) differ between the two species, they are not a useful character for identification as the procedure of evagination of the internal sac is not feasible for many entomologists. However, clear differences in the ratio internal sac to aedeagus length can usually be seen in embedded median lobes of aedeagus (Fig. 39).

The body lengths of *C. javetii* and *C. herbacea* are not a good diagnostic character as the specimens in our collections show a stronger overlap than expected based in Deuve (2011). However, the proportion of the pronotum as described by Matalin and Chikatunov (2016) seems to be a good character for the identification of the two species.

As *C. javetii* has recently been recognized as a species, the specimens from Israel are listed under the species names *C. campestris* or *C. herbacea* (e.g. Nussbaum 1987; Valdenberg 1983). The specimens from South-west Syria are published under the taxon names *herbacea* and *thughurica* (e.g. Avgin and Wiesner 2009). Ptashkovsky (2013) included a photograph of *C. herbacea*, but it is unlikely that this specimen was collected in Israel.

The correct name of the taxon is *javetii* (Chaudoir 1861: 1), not *javeti* (e.g. Deuve 2011; Matalin and Chikatunov 2016).

##### Conservation.

The species is most likely extinct in Israel, as there have been no new records in the last two decades despite intensive searches on the sites from which the species was previously known. In most cases, the relevant habitats have been destroyed. Populations still exist on the Syrian side of the Hermon, as specimens have been collected there as recently as 2007 (<Syria Occ. Bludan / 40 km west of Damascus / 1700–2300m Antilebano(n) / leg. A. Wrzecionko / 5.5.2007> and same locality, but < … 2200m / Skoupý leg.>; CAL, CGS).

#### 3. Cicindela herbacea

Taxon classificationAnimaliaColeopteraCicindelidae

Klug, 1832

##### Habitat.

Unknown. Diurnal.

##### Phenology.

End of March to July (northern Lebanon; CAL, CGS).

##### Distribution range.

From southern Asia Minor to Lebanon, Syria, and Iran (Deuve 2011; 2012). Range overlap with *C. javetii* (Deuve 2011).

##### Distribution in the southern Levant.

No records, but occurrence possible.

##### Taxonomic notes.

see *C. javetii*.

#### 4. Cicindela asiatica

Taxon classificationAnimaliaColeopteraCicindelidae

Audouin & Brullé, 1839

##### Habitat.

Unknown. Most of the records are from mountain areas (Korell 1988).

##### Phenology.

May (CAL).

##### Distribution range.

From Turkey to Iran (Wiesner 1992).

##### Distribution in the southern Levant.

No records.

##### Taxonomic notes.

Two subspecies are known. The nominate form occurs in Syria (Wiesner 1992).

#### 5. Calomera concolor

Taxon classificationAnimaliaColeopteraCicindelidae

(Dejean, 1822)

##### Habitat.

Sandy beaches (Austin et al. 2008). Larvae inhabit the beach from the high water line to the beginning of dunes (Arndt et al. 2005). Diurnal.

##### Phenology.

Main activity period of adults from June to August (Arndt et al. 2005).

##### Distribution range.

Along the coasts of the Mediterranean Sea from Aegean Islands to Syria (including Crete, Cyprus, and southern Turkey) (Austin et al. 2008; Franzen 1999).

##### Distribution in the southern Levant.

No records.

##### Taxonomic notes.

The populations from Cyprus, eastern Turkey and Syria belong to the subspecies rouxi (Barthélemy, 1835) (Franzen 1999).

##### Conservation.

This species is sensitive to disturbances caused by touristic activities on beaches. Even relatively extensive tourism can reduce the activity of adult beetles, and can prevent the development of larvae (Arndt et al. 2005).

#### 6. Calomera fischeri

Taxon classificationAnimaliaColeopteraCicindelidae

(Adams, 1817)

##### Habitat.

On river banks and next to freshwater ponds with sparse vegetation on sandy, sometimes cohesive soil (Arndt 2011; Avgin 2006; Werner 1992). In desert habitats the species can be widespread (cf. Wiesner 1996). Diurnal.

##### Phenology.

In Turkey, adults from the end of May to the beginning of September (Avgin 2006).

##### Distribution range.

from southeastern Europe to central Asia and India, southwards to Turkey and Syria (Acciavatti and Pearson 1989; Werner 1991; Wiesner 1992). Austin et al. (2008: 22) questioned the occurrence on Cyprus. However, Horn and Roeschkle (1891) list the species for Cyprus, and old records exist in SDEI: <Cyprus, Baudi> (3 specimens in the collections of Kraatz and Rottenberg, Lutz Behne, pers. com.) Therefore, the species is listed correctly by Putchkov and Matalin (2003; 2017: 219) for Cyprus.

##### Distribution in the southern Levant.

The distribution range of the species in the Middle East seems to be incorrectly reported. Despite the fact that numerous authors mention the species from Israel (Avgin 2006; Putchkov and Matalin 2003), we do not know of any verified record from the country. There are no specimens in SMNHTAU, and the species is mentioned neither by Nussbaum (1987) nor by Valdenberg (1983). No verifiable records are known from Jordan (Putchkov pers. comm.). However, the species is still listed for Jordan in the latest version of the Palaearctic Catalogue of Coleoptera (Putchkov and Matalin 2017).

In Israel and Jordan, the species’ typical habitats, such as river banks in dynamic floodplains or wet pioneer vegetation with patches of bare ground, have mostly been destroyed or are strongly influenced by human activity. Therefore, a recent occurrence of *C. fischeri* in the Mediterranean part of the southern Levant is unlikely. There is a small chance that the species can be found in wadis or close to water reservoirs in the desert regions (cf. Wiesner 1996).

##### Taxonomic notes.

The nominate subspecies occurs in the northern Levant, while the subspecies elongatosignata (W. Horn, 1922) is found on the Arabian Peninsula (Wiesner 1992).

#### 7. Calomera alboguttata

Taxon classificationAnimaliaColeopteraCicindelidae

(Klug, 1832)

##### Habitat.

In riverbeds with gravel banks and stones, or on sandy ground close to water (Werner 2000).

##### Phenology.

Unknown.

##### Distribution range.

Northeast Africa and the Arabian Peninsula (Werner 2000). Horn (1931) already questioned the validity of the record from Port Said. Not listed by Alfieri (1976) for Egypt.

##### Distribution in the southern Levant.

No record. The nearest known population is found in Wadi Sharis (Abdel-Dayem et al. 2003).

#### 8. Calomera aulica

Taxon classificationAnimaliaColeopteraCicindelidae

(Dejean, 1831)

##### Habitat.

Mainly in salty habitats, such as sea shores and marshlands with salt crusts, or rocky habitats (Abdel-Dayem 2004; Horn 1931; Werner 1991; 2000). Diurnal.

##### Phenology.

On the Sinai Peninsula from February until October (Abdel-Dayem et al. 2003), in the Dead Sea region from May to December (Matalin and Chikatunov 2016: 120; Nussbaum 1987).

##### Distribution range.

From Senegal through northern Africa and Greece to the Middle East and Pakistan (Acciavatti and Pearson 1989; Arndt 2011; Horn 1931; Werner 2000).

##### Distribution in the southern Levant.

In northern and southern Sinai along the coasts of the Mediterranean and of the Red Sea, and along the Suez Canal. In Israel in the Dead Sea region (Abdel-Dayem et al. 2003; Matalin and Chikatunov 2016; Nussbaum 1987). Rittner (pers. comm.) found a population in the vicinity of Akko on a rocky beach (documented by photographs, see the homepage Israel-nature-site 2017). The only known records from Jordan date back to the 1940s (Matalin and Chikatunov 2016; 4 specimens in SMNHTAU). Now also recent records from Jordan: “JOR-at-Tafila, Hammam Afra, Hot Springs, 08.05.2010” (CGD), “Dead Sea, Wadi ‘Atun, N Wadi Mujib, same date” (CSH).

##### Taxonomic notes.

The coloration can be useful for distinguishing some *Calomera* species, especially *C. aulica*, *C. diania*, *C. littoralis* and *C. aulicoides* (Arndt 2011). Nevertheless, *C. aulica* is extremely variable in color, and the coloration of the elytra ranges from black to bronze or copper with a aditional colors also occurring.

**Figure 40.**
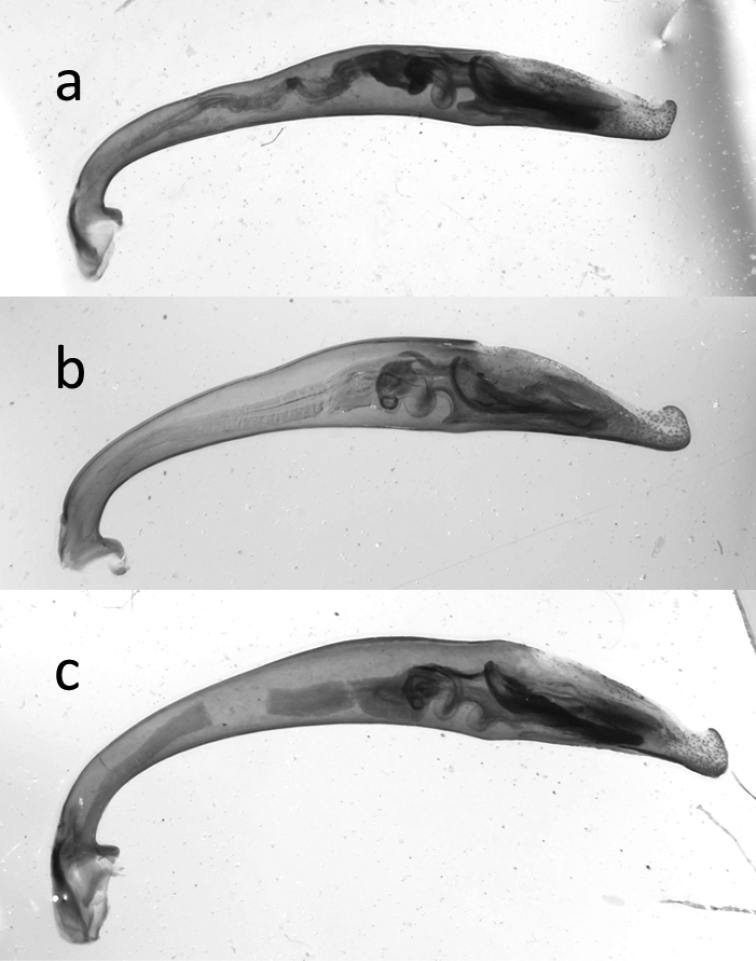
Median lobes of the aedeagus of species of the *Calomera aulica* group: **a** *C. aulica* **b** *C. diania* **c** *C. aphrodisia*.

**Figure 41.**
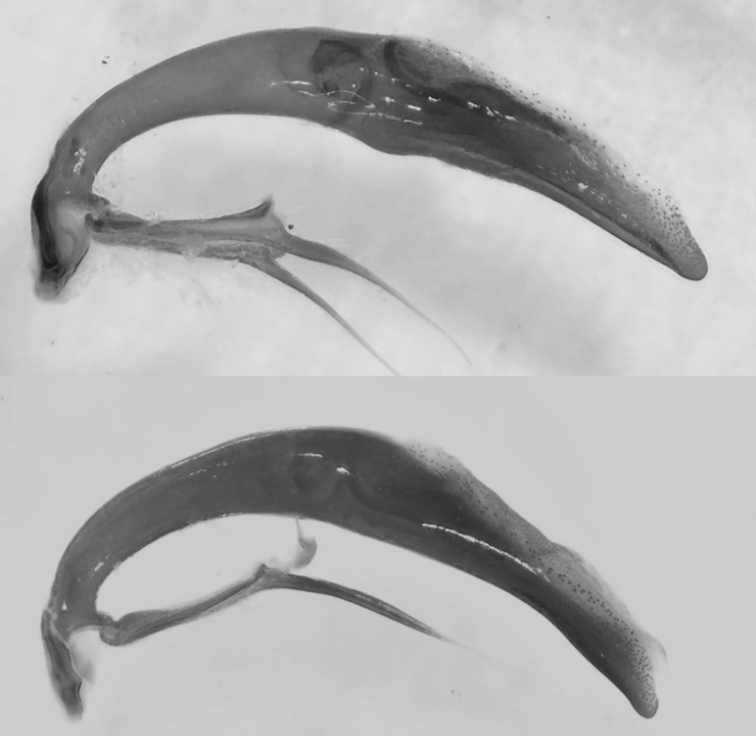
Median lobes of the aedeagus of species of *Calomera aulicoides* (above) and *C. littoralis winkleri* (below).

The pale elytral pattern of *C. aulica* is similar to that of *C. aulicoides*. Although the tip of the copulatory pieces of median lobe of aedeagus is similar in both species, they can be easily distinguished from each other by the external shape of the aedeagus (Figs 40, 41). A reliable character for differentiating *C. aulicoides* from related species is the number of teeth on the inner side of the left mandible: *C. aulicoides* has 4 teeth, while *C. aulica* and *C. littoralis* have only 3 (Matalin and Chikatunov 2016) (Figs 51, 52).

##### Conservation.

Rare and endangered in Israel. Few records exist from recent decades.

#### 9. Calomera diania

Taxon classificationAnimaliaColeopteraCicindelidae

(Tschitschérine, 1903)

##### Habitat.

Freshwater habitats. In contrast to *C. aulica*, which can occur on both coastal and inland habitats, *C. diania* is an exclusive inland species (Naviaux 1983).

##### Phenology.

End of February to August (Naviaux 1983, ZISP, CGD)

##### Distribution range.

From Iraq to Pakistan and the southern Arabian Peninsula (Wiesner 1992).

##### Distribution in the southern Levant.

No record.

##### Taxonomic notes.

Naviaux (1983) gave an excellent description with which to check any potential records from the Levant.

#### 10. Calomera aphrodisia

Taxon classificationAnimaliaColeopteraCicindelidae

(Baudi di Selve, 1864)

##### Habitat.

Rocky habitats in the littoral zone (Austin et al. 2008; Aydın 2011c; Franzen 2001a; Horn 1931). Larval development also occurs in this habitat (Lisa 2002).

##### Phenology.

May to August (Franzen 2001a; database Gebert; Lisa 2002).

##### Distribution range.

From Sicily and Greece to Turkey and Syria (Wiesner 1992).

##### Distribution in the southern Levant.

The first verifiable record from Israel was found by the cicindelid expert A. Putchkov (pers. comm.). He saw an old specimen from northern Israel (label information: <Izrael: Khaifa env.>), together with an old record from Syria (label information: <N Syrien, Ladyk env.); both specimens are preserved in ZISP. No recent records from Israel, but suitable habitats still exist in northern Israel (e.g. close to Akko and to Hadera).

##### Conservation.

Declining in Turkey due to touristic activities on the beaches (Aydın 2011c; Aydın et al. 2005). As the species occurs only locally and in habitats which tend to be under strong human pressure, the species should be classified at least as threatened. Data are deficient for the southern Levant.

##### Taxonomic notes.

Three subspecies are known, with the nominate form occurring in Turkey and in Syria (Wiesner 1992).

#### 11. Calomera littoralis (Fabricius, 1787) , ssp. winkleri

Taxon classificationAnimaliaColeopteraCicindelidae

(Mandl, 1934)

##### Habitat.

A coastal species which colonizes both sea shores with sandy or with cohesive soils as well as salty marshlands found behind the dunes, especially those covered with salt crusts during the summer and where the vegetation is dominated by *Anthrocnemum* species and by *Tamarix tetragyna*. Also found in river mouths and in freshwater habitats (Austin et al. 2008; Nussbaum 1987; Valdenberg 1983; own observations). Diurnal.

##### Phenology.

Middle of February until November (Matalin and Chikatunov 2016; Nussbaum 1987).

##### Distribution range.

From Greece to Iran and Central Asia; southwards to Israel (Mandl 1981b).

##### Distribution in the southern Levant.

Along the Mediterranean coast from the mouth of Nahal Betzet (=Nakhal Bezet) to the Gaza strip (Matalin and Chikatunov 2016; Nussbaum 1987). It probably also occurs on the northern coast of Sinai Peninsula, but Abdel-Dayem et al. (2003) did not list the subspecies or the nominate form from Egypt. *Calomera littoralis winkleri* is listed by Puchkow and Matalin (2017) and Nasir and Katbeh-Bader (2017) for Jordan, but not by Matalin and Chikatunov (2016). Nasir and Katbeh-Bader (2017) indicate the species from Ma’in Falls, a typical habitat for *C. aulicoides*, from where they mention also *C. littoralis*. We do not know of any verifiable record for Jordan.

##### Taxonomic notes.

The subspecies winkleri can be differentiated from the other subspecies of *littoralis* using the form of the copulatory piece of the median lobe of the aedeagus (Korell 1988; Mandl 1934; 1981b). Some populations from the eastern part of the distribution range have copulatory pieces which show an intermediate shape between those of *nemoralis* (Olivier, 1790) and *winkleri*. These populations are most probably transitional, and likely are hybrid populations. Nonetheless, they are described as a separate subspecies, *mandli* Mandl, 1934 (Korell 1988; Mandl 1981b).

See also *C. aulica* for further diagnostic characters.

##### Conservation.

Not threatened. Although the species lives along seashores which tend to be intensively influenced by touristic activities, the species has not declined as strongly as other littoral tiger beetles (for Greece: Gebert 2013, for Israel: own observation).

#### 12. Calomera aulicoides

Taxon classificationAnimaliaColeopteraCicindelidae

(J.R. Sahlberg, 1913), stat. rest.

##### Habitat.

On sandy and stony banks close to freshwater (the Jordan River, Sea of Galilee), especially in wadis. Also found in salty habitats close to the Dead Sea. Diurnal.

##### Phenology.

Throughout the year (records from February to December) (Nussbaum 1987, own observations).

##### Distribution range.

From Egypt and southern Turkey to Iran (Cassola 1999; Korell 1988; Mandl 1981b).

##### Distribution in the southern Levant.

In Israel and Jordan along the Jordan Valley from the Hula Valley and the Sea of Galilee to the Dead Sea region, and in the Arava Valley. In Sinai on the Mediterranean Coast and in South Sinai (Abdel-Dayem et al. 2003; Matalin and Chikatunov 2016; Nussbaum 1987).

##### Taxonomic notes.

In the past most authors ranked this taxon as a subspecies (or even as a form with a rank below the subspecies) of *littoralis* (or of another taxon of this species group) (e.g. Mandl 1934). However, later Mandl (1981b) ranked *aulicoides* as a species, though only few authors accepted this ranking (e.g. Korell 1984; Werner 1991), and the majority rank it as a subspecies (Cassola 1999; Matalin and Chikatunov 2016; Putchkov and Matalin 2003, 2017; Wiesner 1992). Pesarini and Monzini (2010: 10) are, to our knowledge, the only authors from the last years, who ranked *aulicoides* as a valid species. However, the authors seem to have confused it with *Calomera aphrodisia* (Baudi, 1864), which occurs in Sicily (Brandmayr et al. 2005; Lisa 2002; Vigna-Taglianti 1993), but is not listed by Pesarini and Monzini (2010).

In the southern Levant, both *littoraliswinkleri* and *aulicoides* occur. They live in sympatry in the north of Israel (in the Hula Valley: see records for *C. littoralis winkleri* published by Matalin and Chikatunov 2016, and own records of *C. aulicoides* from Nahal Guvta (= Wadi al-Hashabi, in some maps indicated as Wadi Guyta; close to the Banias, CAL), while further southwest they are (at least) parapatric with a distance of about 20 km (Tamra – Eilabun) between populations. Although both taxa are flight-active, no intermediate forms are known (in contrast to the form of the copulatory pieces in the *littoralis* subspecies, see above). *Calomera aulicoides* has a clear and easily accessible character for differentiation from *C. littoralis*. While the latter one usually has three teeth on the inner side of the left mandible, *C. aulicoides* has four of them (Figs 51 and 52; Matalin and Chikatunov 2016). Sometimes specimens of *C. littoralis winkleri* have a small fourth tooth on the inner edge of the left mandible (Fig. 52). However, these specimens do not represent hybrid populations as the copulatory piece of the median lobe of aedeagus does not show any intermediate characters. The lack of intermediate populations and the sympatric and parapatric distribution ranges in the southern Levant give evidence for the species status of both, *C. aulicoides* and *C. littoralis*.

It is possible that both taxa occur sympatrically in Jordan as well (Ma’in Falls, Nasir and Katbeh-Bader 2017). We do not know any population of *C. littoralis* from Jordan.

**Figure 42.**
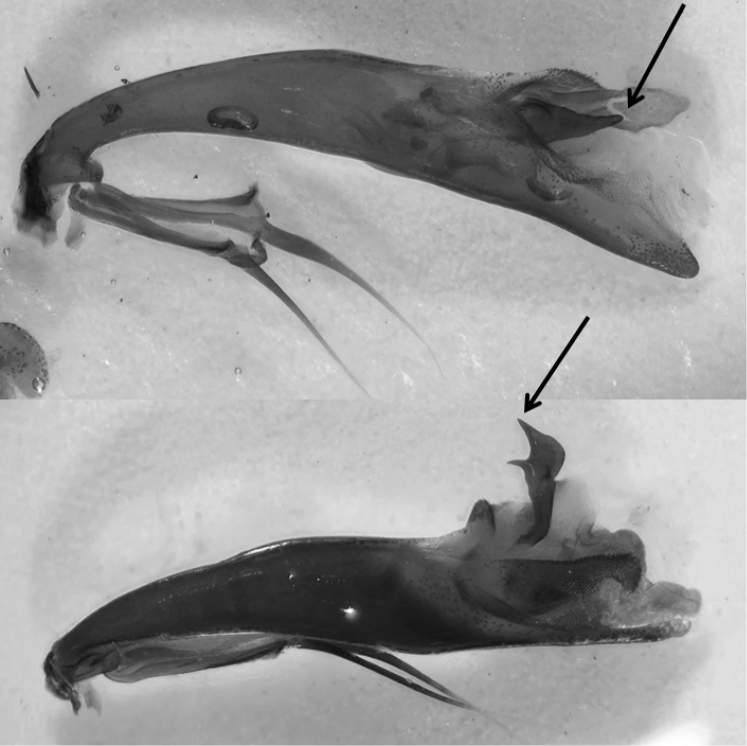
Copulatory pieces (arrows) of the median lobes of aedeagi of *Calomera aulicoides* (above) and *C. littoralis winkleri* (below).

The external shapes of the median lobes of the aedeagi of both taxa do not differ strongly from each other (Fig. 40), though the copulatory pieces are strongly differentiated (Fig. 42). The sharp spines of the copulatory piece in these taxa may even act in a similar way to the spines of the endophallus in closely related Carabus species of the subgenus Ohomopterus, which seem to be an example of lock-and-key genitalia (Sota and Kubota 1998). This is in contrast to most other animals, as in depth discussed by Eberhard (2010; 1985). Caution is necessary in postulating lock-and-key-functions for the genitalia in tiger beetles as we lack empirical evidence.

**Figure 43.**
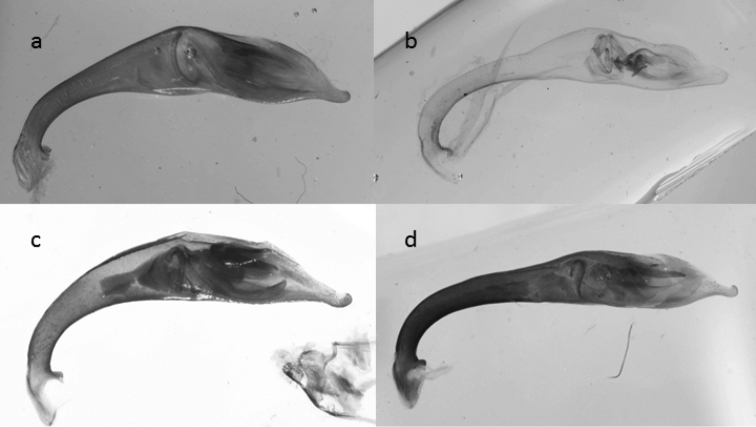
Median lobes of the aedeagus of *Cephalota* species: **a** *C. littorea* **b** *C. tibialis* **c** *C. circumdata* **d** *C. vartianorum*.

Moreover, we have to be cautious to establish species ranks solely based on the external shape of the median lobe of aedeagus. In this respect, clearly differentiated taxa of ground beetles can have an excessive geneflow (e.g. Matern et al. 2011).

The change in the taxonomic rank of *C. aulicoides* stat. rest. indicates the need for a revision of the entire *C. littoralis* group. As a recent phylogeographic study from southeastern Europe reveals, molecular approaches can help to solve the taxonomic chaos in this group with its overlooked or neglected taxa (Jaskuła et al. 2016).

See also *C. aulica* for further diagnostic characters.

##### Conservation.

Not threatened in Israel or Jordan. Still widely distributed in the Sea of Galilee region and in the wadis around the Dead Sea, also in strongly grazed habitats.

#### 13. Calomera fimbriata

Taxon classificationAnimaliaColeopteraCicindelidae

(Dejean, 1831)

##### Habitat.

Sandy and stony river banks and on the shore of lakes (Werner 2000). See also *Habrodera nilotica*.

##### Phenology.

In Africa throughout most of the year (Werner 2000).

##### Distribution range.

From Senegal to Sudan and Ethiopia (Werner 2000). Probably does not occur in Egypt (see discussion in Abdel-Dayem et al. 2003).

##### Distribution in the southern Levant.

No record.

##### Taxonomic notes.

Described from Ambukol (= Ambukohl) which belongs today to Sudan (Abdel-Dayem et al. 2003).

#### 14. Habrodera nilotica

Taxon classificationAnimaliaColeopteraCicindelidae

(Dejean, 1825)

##### Habitat.

Freshwater habitats (Abdel-Dayem 2004). Sandy and stony banks of rivers and lakes, especially on yellow sand. In Africa it frequently occurs together with *C. fimbriata* (Werner 2000). Diurnal and nocturnal. Attracted by light.

##### Phenology.

In Africa and in the Middle East throughout almost the entire year (Matalin and Chikatunov 2016; Werner 2000). In Egypt records from January, June, August and November (Alfieri 1976).

##### Distribution range.

Widely distributed in Africa: From Senegal to Somalia and from Egypt to South Africa (Werner 2000).

##### Distribution in the southern Levant.

Wadi Isla (southern Sinai) (Abdel-Dayem et al. 2003; Alfieri 1976). Chikatunov et al. (2006) published records from Israel. We could not find any verifiable record from this survey in SMNHTAU. Therefore, the occurrence of this species in Israel is highly questionable.

#### 15. Cephalota (Taenidia) littorea

Taxon classificationAnimaliaColeopteraCicindelidae

(Forskål, 1775) [sic]

##### Habitat.

On seashores and in marshland habitats. Diurnal and nocturnal. Attracted by light (Abdel-Dayem et al. 2003; Cassola 1972; Nussbaum 1987).

##### Phenology.

May to September (Abdel-Dayem et al. 2003; Nussbaum 1987).

##### Distribution range.

From southern Spain to the Arabian Peninsula and Northeast Africa (Gebert 1991).

##### Distribution in the southern Levant.

Only in southern Sinai (Abdel-Dayem et al. 2003; Alfieri 1976; Gebert 1991; Nussbaum 1987). No record from Israel or Jordan (Putchkov and Matalin 2017), but populations still exist not far from the border to both countries (<Bir Suweir / Sinai 30.4.2016 / A. Gera> SMNHTAU, CAL).

##### Taxonomic notes.

Only the nominate subspecies occurs in the southern Levant (Gebert 1991). *Cephalota littorea* and *C. tibialis* have long been confused (e.g. Mandl 1935). The revision of Gebert (1991) revealed the species status of both taxa, and described their variability (incl. genitalia and pale coloration pattern on elytra). The subspecies *C. littorea alboreductata* (Horn, 1934) occurs south of the distribution range of *C. littorea* s.str. (Gebert 1991).

Although the taxon *goudotii* (Dejean, 1829), which occurs along the coasts of the western Mediterranean, is currently ranked as a subspecies of *C. littorea*, it is probably a valid species. *Cephalota littorea* s.str. and *goudotii* do not occur parapatrically as their ranges are separated from each other by a gap which is partially filled by the distribution range of *tibialis*. Moreover, the differences in the median lobe of the aedeagus (shape, internal sac) may support the species status of both *goudotii* and *littorea* (but see *C. aulicoides* for discussion of genital structures as characters to delineate species).

The correct spelling of the author name is Forskål (Forskål 1775) and not Forsskål (Putchkov and Matalin 2017).

#### 16. Cephalota (Taenidia) tibialis

Taxon classificationAnimaliaColeopteraCicindelidae

(Dejean, 1822)

##### Habitat.

Shorelines of salt lakes and ponds (Austin et al. 2008; Jaskuła and Rewicz 2015; Lisa 2002), sandy beaches (Nussbaum 1987). Nocturnal (Abdel-Dayem et al. 2003). Attracted by light (Nussbaum 1987).

##### Phenology.

February to September (Abdel-Dayem et al. 2003).

##### Distribution range.

From Tunisia to Egypt (Gebert 1991).

##### Distribution in the southern Levant.

along the Mediterranean coast of the Sinai Peninsula (Gebert 1991). No record from Israel (Matalin and Chikatunov 2016; Nussbaum 1987).

##### Taxonomic notes.

Only the nominate subspecies in the southern Levant, the other two subspecies in northern Africa and on Cyprus (Gebert 1991). See also *C. littorea*.

#### 17. Cephalota (Taenidia) circumdata

Taxon classificationAnimaliaColeopteraCicindelidae

(Dejean, 1822)

##### Habitat.

On salty habitats which have very sparse vascular plant vegetation. Often found on salty crusts of lagoons and ponds behind the coastal dunes (Lisa 2002), but can also be found in similar habitats farther inland (Cassola 1970; Franzen 1996). Diurnal and nocturnal species. Attracted by light.

##### Phenology.

In Italy from June to October with activity maximum in June and July (Lisa 2002).

##### Distribution range.

A Mediterranean species from Spain and Algeria to Turkey (Cassola 1970; Lisa 2002).

##### Distribution in the southern Levant.

No verified population. – The occurrence in El Tor (southern Sinai) has been questioned by Horn and Roeschke (1891). Schatzmayr (1936) could not examine specimens from there or from anywhere else on the peninsula. Alfieri (1976) and Abdel-Dayem et al. (2003) and Abdel-Dayem (2004) list the species for southern Sinai, while Nussbaum (1987) and Matalin and Chikatunov (2016) do not. Horn et al. (1990) report that Alfieri’s beetle collection has been incorporated into the collection of Frey, which is now preserved in the natural history museum in Basel. However, no verifiable specimens of *C. circumdata* from Sinai are preserved in the Frey collection, and only few tiger beetle individuals from Alfieri’s collection are found in Basel (Sprecher-Uebersax, pers. comm.). We do not know of any verifiable record from the Sinai. As all other populations are known from areas with a typical Mediterranean climate, we believe that *C. circumdata* is not found in the Sinai (cf. Matalin and Chikatunov 2016).

##### Taxonomic notes.

*Cephalota circumdata* has several subspecies which are mainly characterized by the elytral pale patterns. However, Franzen (1996) reported strong pattern variability within some populations.

#### 18. Cephalota (Taenidia) vartianorum

Taxon classificationAnimaliaColeopteraCicindelidae

(Mandl, 1967)

##### Habitat.

Saline habitats with sparse vegetation and salt crusts during summer. Diurnal and nocturnal. Attracted by light (Korell 1984).

##### Phenology.

Spring, records from February to June (Gebert 2016; Matalin and Chikatunov 2016; Nussbaum 1987).

##### Distribution range.

Israel, Syria to Iran (Gebert 2016).

##### Distribution in the southern Levant.

In the Dead Sea region of Israel (Gebert 2016; Matalin and Chikatunov 2016; Nussbaum 1987). We do not know any verifiable record from Jordan. This is in agreement with the distribution indications of Puchkov and Matalin (2003) and Wiesner (1992), but it is in disagreement with Puchkow and Matalin (2017). The country indications for Saudia-Arabia, Yemen and Jordan have not been verified (Matalin pers. comm. to Jörg Gebert on November 26, 2017).

##### Taxonomic notes.

While in older publications this taxon is listed as a subspecies of *C. zarudniana* (Tschitschérine, 1903), Gebert (2016) elevated it to full species rank. *Cephalota vartianorum* differs from *C. zarudniana* by slightly slender habitus as well as shape of the median lobe of aedeagus, and in the complete lack of white setae on the genae.

##### Conservation.

Critically endangered in Israel. Israel has a national responsibility for the worldwide conservation of the taxon.

Tiger beetles of coastal habitats are often sensitive to touristic use of beaches (Aydın et al. 2005; U.S. Fish and Wildlife Service 2009). Most of the Israeli beaches known to host this species are intensively used as recreational areas. With high probability at least some of the (local) populations have become extinct. Matalin and Chikatunov (2016) stated that the most recent records date from the late 80’s to the 90’s of the last century. Our most recent records are from 1990 in Israel (Neot HaKikkar = Neot Hakikar, 13. May 1990, leg. E. Orbach, COQ, CAL) and from 2000 in Syria (Euphrates, database Gebert). All other 34 entries in the database Gebert date back to the late 1980s and 1990s. Intensive searches, including use of light traps at night, of several sites in Israel such as the Enot Tsukim Reserve (= Enot Zuqim = Enot Zukim = Einot Zukim = En Fescha) from where populations have been previously recorded, have yielded no new records. The lowering of the water table and changes in land use in the Dead Sea region have strongly impacted many habitats, both freshwater and saltwater. Therefore, at further studies of the Dead Sea region, both on the Israeli and the Jordanian side, are needed to establish wether or not populations still exist.

#### 19. Homodela ismenia

Taxon classificationAnimaliaColeopteraCicindelidae

(Gory , 1883)

##### Habitat.

In open forests and in grasslands, mainly on sandy ground in higher elevations (Avgin 2006; Korell 1988).

##### Phenology.

Spring species (Korell 1988).

##### Distribution range.

Turkey and Syria (Franzen 2003; Wiesner 1992).

##### Distribution in the southern Levant.

No records.

#### 20. Cylindera (Eugrapha) contorta (Fischer von Waldheim, 1828) , ssp. valdenbergi

Taxon classificationAnimaliaColeopteraCicindelidae

(Mandl, 1981)

##### Habitat.

In the southern Levant found exclusively in sandy shore habitats along the Mediterranean Sea. Larval development takes place a few centimeters above the mean sea level, just above where most of the waves break (Valdenberg 1983), though larval holes are occasionally flooded by seawater. The nominate subspecies is found in both coastal and inland habitats (Cassola and Jaskuła 2004). Attracted by light (own observation).

##### Phenology.

Adults from the beginning of May until mid-November (Valdenberg 1983). Few adults also from March onwards (Matalin and Chikatunov 2016; Nussbaum 1987). The larvae overwinter mostly as second and third instars (and rarely also first instars). Egg laying begins immediately with the appearance of the adults in spring, and in certain years a few individuals may complete an entire life-cycle in the same summer, though most do not (Valdenberg 1983).

##### Distribution range.

The nominate form is found from south-eastern Europe (Romania and Ukraine) and Asia Minor to Central Asia and China. The  subspecies valdenbergi is patchily located in a small area along the Mediterranean coast from western Egypt to northern Israel (Horn 1931, Mandl 1981). The nominate form does not occur along the Mediterranean coast (Wiesner 1992).

##### Distribution in the southern Levant.

In Egypt around Abu Qir, Maadia and Ras el Bar (Abdel-Dayem et al. 2003; Alfieri 1976; Horn 1931; Mandl 1981a; Nussbaum 1987). In Israel from Bat Yam to Akko, though the Bat Yam population is thought to be extinct since several decades (Valdenberg 1983; own observation).

##### Taxonomic notes.

Mandl (1981a) described the taxon *valdenbergi* from Ma’agan Michael as a subspecies. It is characterized by the excessive pale elytral pattern (Figs 31, 32), though there is some variation in the pattern between individuals. However, the pale elytral coloration differs even within the nominate form strongly. Some populations from the Caspian Sea show similar elytral pattern as *valdenbergi* (cf. Mandl 1981a; Werner 1992). Mandl mentioned in his description small differences in the copulatory pieces of the median lobe of aedeagus. As we believe this character is still not sufficiently studied. But the material we studied let us assume that *valdenbergi* differs from the nominate subspecies at least in the proportion and shape of the pronotum and the elytra (Fig. 29). We recommend conducting a detailed study, including morphometric and molecular methods, to clarify the status of both taxa. Ptashkovsky (2013) includes a photograph of “*Lophyra contorta valdenbergi*”, but the shown specimen belongs to the nominate subspecies of *Cylindera contorta* (cf. Fig. 29), and with all probability the photographed specimen was not collected in Israel.

**Figure 44.**
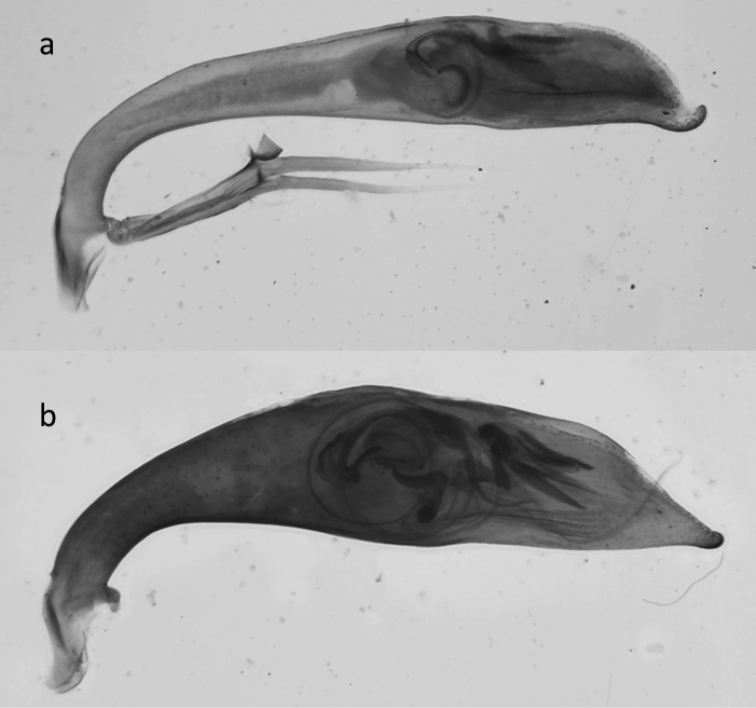
Median lobes of the aedeagus of *Cylindera* species: **a** *C. contorta* s.str. **b** *C. rectangularis*.

##### Conservation.

Critically endangered in Israel. Tiger beetles of coastal habitats tend to be very sensitive to touristic use of beaches (Aydın et al. 2005; U.S. Fish and Wildlife Service 2009). Most of the Israeli beaches which are known to host this species are intensively used as recreational areas, and therefore at least many of the populations have gone extinct. As a metapopulation structure may be possible in this species, the decrease in some populations can have a tremendous effect on the survival of the entire subspecies. Matalin and Chikatunov (2016) stated that the latest records date from the late 80s to the 90’s of the last century, and our most recent records are from 2003 (Shefeh Na’aman Nature Reserve, COQ). As the entire world population of this taxon is located in Israel (the majority of the known populations) and in Egypt, these countries bear the responsibility for the worldwide preservation of this subspecies.

#### 21. Cylindera (Ifasina) rectangularis

Taxon classificationAnimaliaColeopteraCicindelidae

(Klug, 1832)

##### Habitat.

Banks of freshwater in wadis (Abdel-Dayem and Kippenhan 2013), especially on loamy soil (Werner 2000). In Saudi Arabia it co-occurs with *Calomera aulica*, *C. alboguttata* and *Myriochila melancholica* (Abdel-Dayem and Kippenhan 2013).

##### Phenology.

March, but more frequently in June (Abdel-Dayem and Kippenhan 2013).

##### Distribution range.

From Central Africa to Sudan and Saudi Arabia (Abdel-Dayem and Kippenhan 2013).

##### Distribution in the southern Levant.

No record.

#### 22. Myriochila (s.str.) melancholica

Taxon classificationAnimaliaColeopteraCicindelidae

(Fabricius, 1798)

##### Habitat.

Margins of both stagnant and running freshwater bodies, including artificial water reservoirs, and in salty habitats (Austin et al. 2008; Jaskuła and Rewicz 2015; Lisa 2002). Diurnal. Attracted by light (Abdel-Dayem et al. 2003).

**Figure 45.**
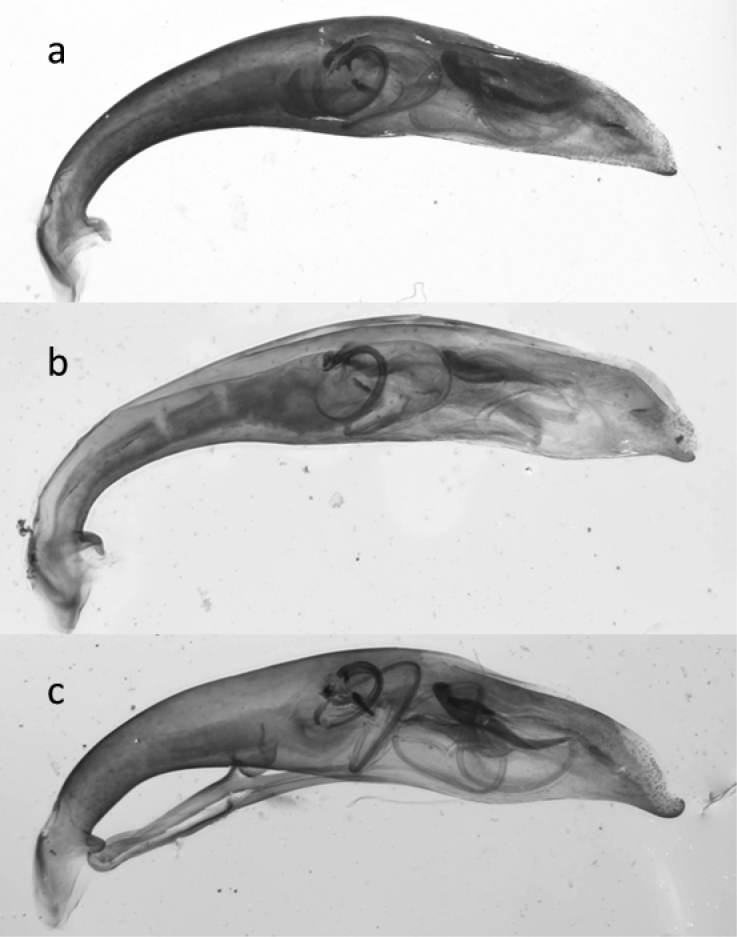
Median lobes of the aedeagus of *Lophyridia* species: **a** *L. flexuosa* **b** *L. hilariola* **c** *L. histrio*.

**Figure 46.**
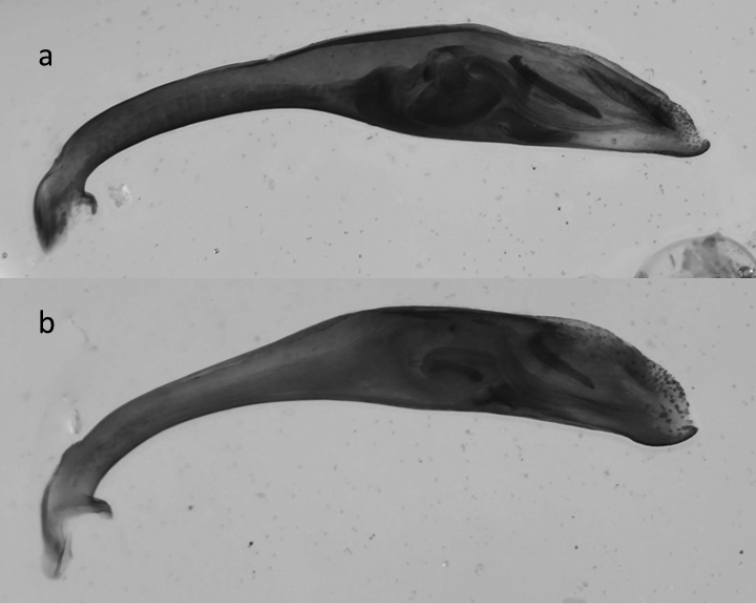
Median lobes of the aedeagus of *Myriochila* species: **a** *M. melancholica* **b** *M. orientalis*.

##### Phenology.

March to December (Abdel-Dayem et al. 2003; Matalin and Chikatunov 2016; Nussbaum 1987).

##### Distribution range.

From southern Europe to southern Africa and from the Cape Verde Islands to China (Wiesner 1992).

##### Distribution in the southern Levant.

Widespread in Sinai, Israel and Jordan (Matalin and Chikatunov 2016; Nasir and Katbeh-Bader 2017; Wiesner 1992).

##### Conservation.

Not endangered, it is abundant even in habitats strongly influenced by human activity (e.g. on intensively grazed sites or on wet fallow land close to Tel Aviv and Amman).

#### 23. Myriochila (Monelica) orientalis

Taxon classificationAnimaliaColeopteraCicindelidae

(Dejean, 1825)

##### Habitat.

Unknown.

##### Phenology.

Unknown.

##### Distribution range.

From Turkey and Syria to China (Wiesner 1992).

##### Distribution in the southern Levant.

No record.

#### 24. Myriochila (Monelica) dorsata

Taxon classificationAnimaliaColeopteraCicindelidae

(Brullé, 1834)

##### Habitat.

Semi-desert and savanna habitats (Werner 2000).

##### Phenology.

Unknown.

##### Distribution range.

Southern Sahel zone from Mauritania and Senegal to Sudan (Werner 2000). Listed by Horn and Roeschke (1891) also for Egypt. Horn (1931) knew of four specimens labelled “Egypt”, all from different collections. Therefore it seems unlikely that all records are mislabelled. Nonetheless, the records may refer to Egypt in its historical sense which includes parts of modern-day Sudan (cf. Alfieri 1976).

##### Distribution in the southern Levant.

No record.

#### 25. Hypaetha singularis

Taxon classificationAnimaliaColeopteraCicindelidae

(Chaudoir, 1876)

##### Habitat.

Sandy seashores (Abdel-Dayem et al. 2003).

##### Phenology.

Recorded in Egypt from May to August (Abdel-Dayem et al. 2003, Matalin and Chikatunov 2016). In SMNHTAU are also specimens collected in September (own observation).

**Figure 47.**
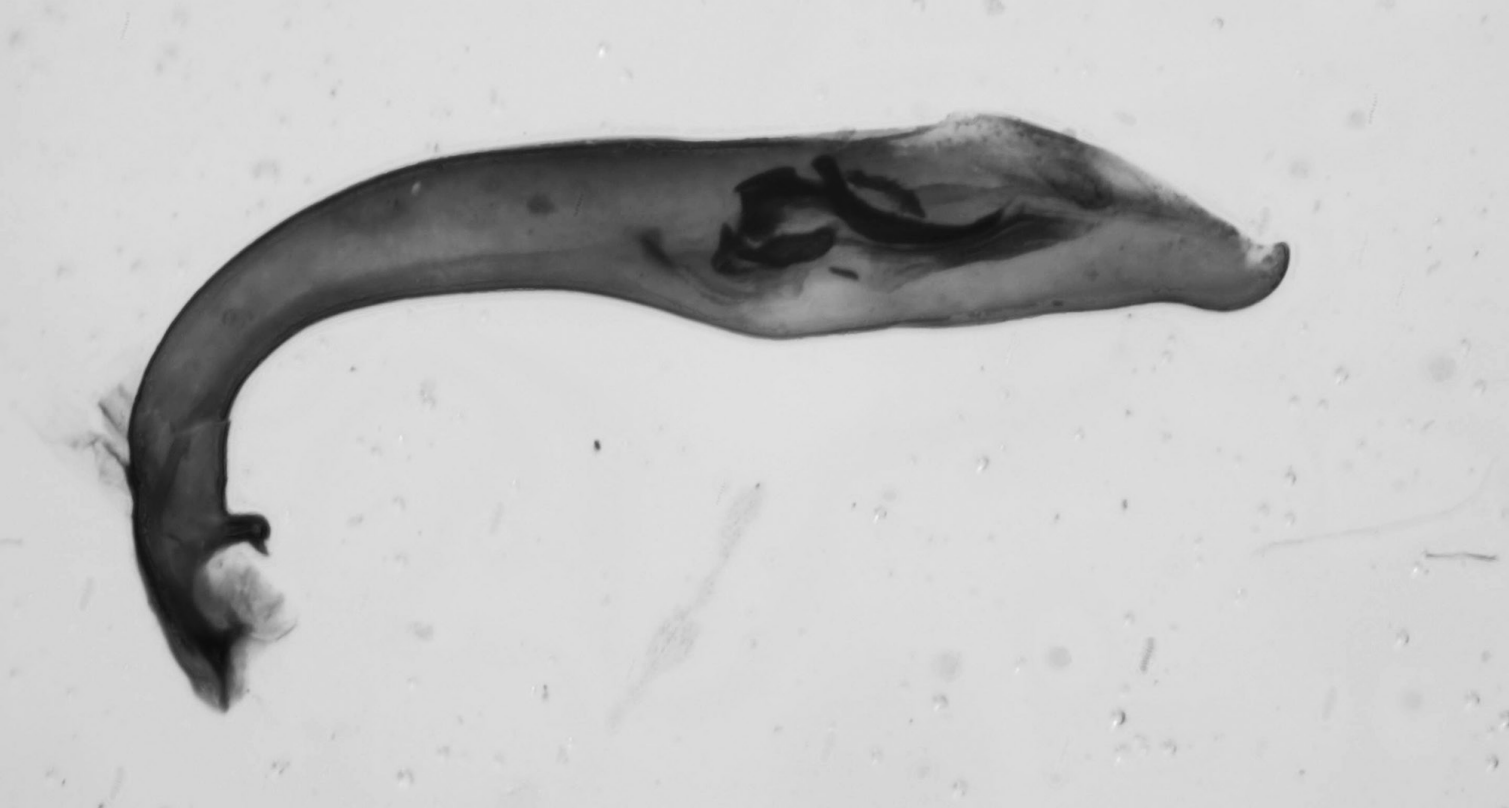
Median lobe of the aedeagus of *Hypaetha singularis*.

**Figure 48.**
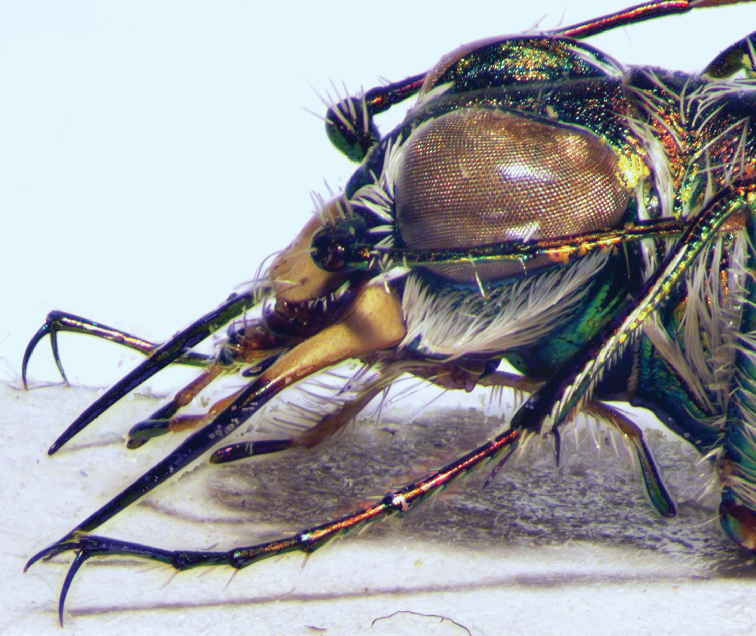
Head in lateral view: *Calomera alboguttata* (above) and *Habrodera nilotica* (below).

**Figure 49.**
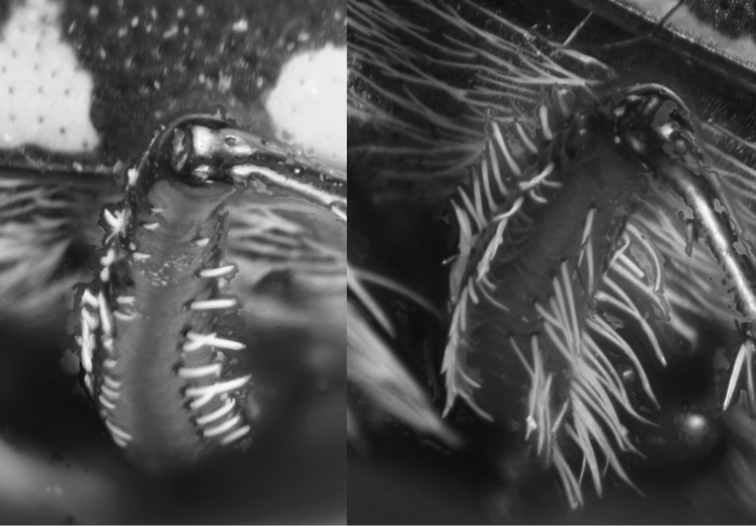
Metafemora, lateral view on lower side: *C. aulica* (left), *C. aulicoides* (right).

**Figure 50.**
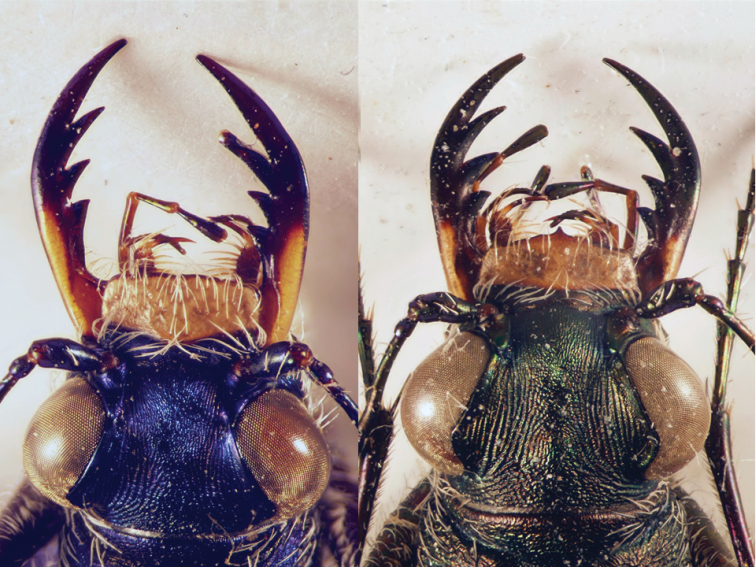
Head of *Calomera* species in dorsal view: *C. diania* (left), *C. aulica* (right).

**Figure 51.**
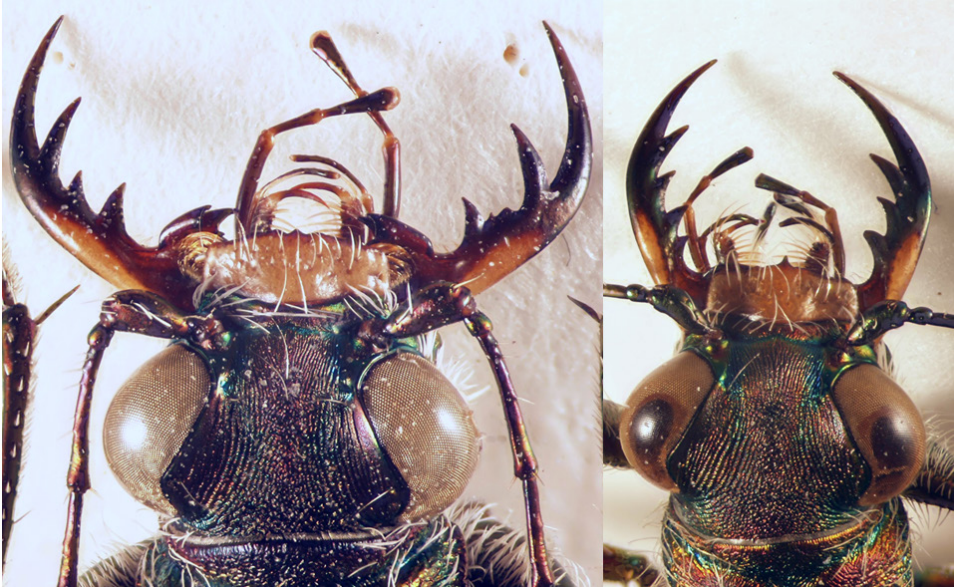
Head of *Calomera* species in dorsal view: *C. aphrodisia* (left), *C. aulicoides* (right).

**Figure 52.**
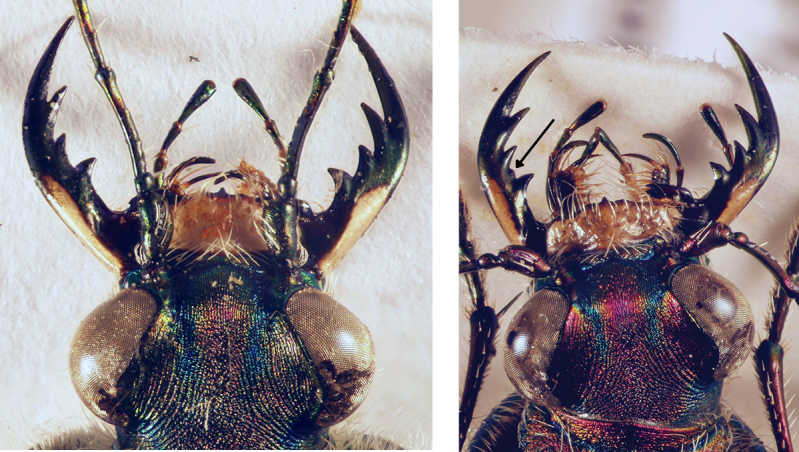
Head of *Calomera littoralis winkleri*: with regular form of left mandible (left) and a small fourth tooth on the inner side of left mandible (arrow, right).

**Figure 53.**
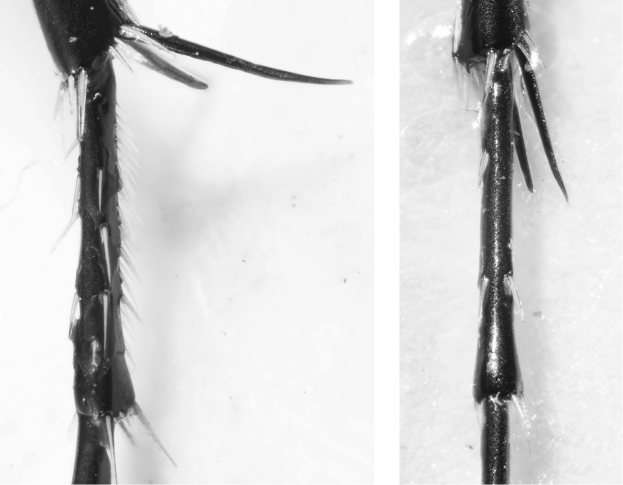
Tibial spurs and 1^st^ tarsal segment of *Calomera littoralis winkleri* (left) and *Calomera aulicoides* (right).

**Figure 54.**
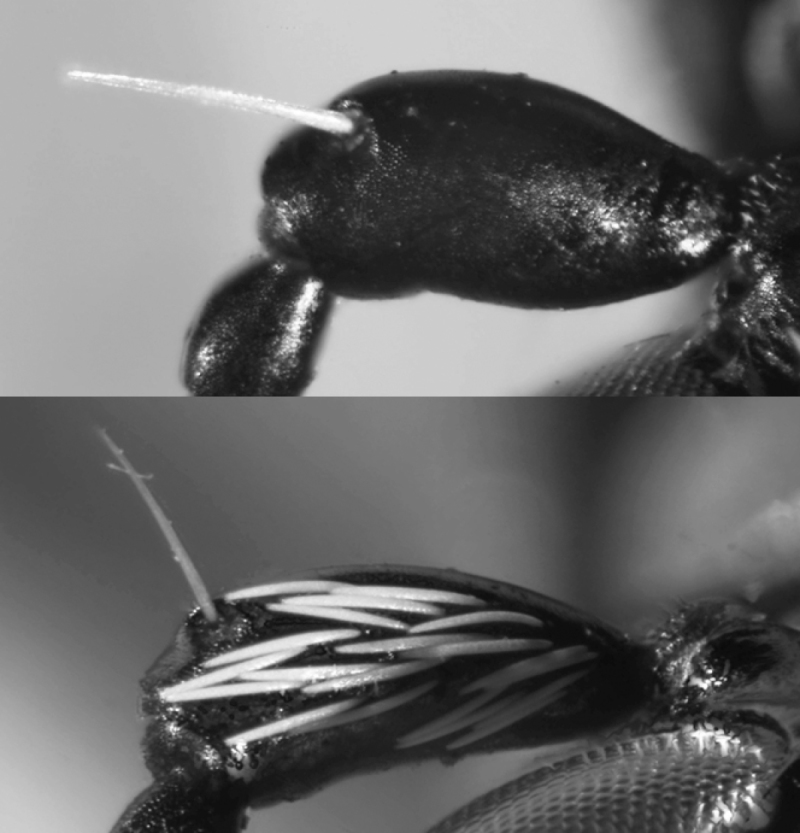
1^st^ antennal segment: with only one erect distal seta (*Calomera alboguttata*; above) and with one erect distal seta and additional recumbent white setae (*Lophyra histrio*; below).

**Figure 55.**
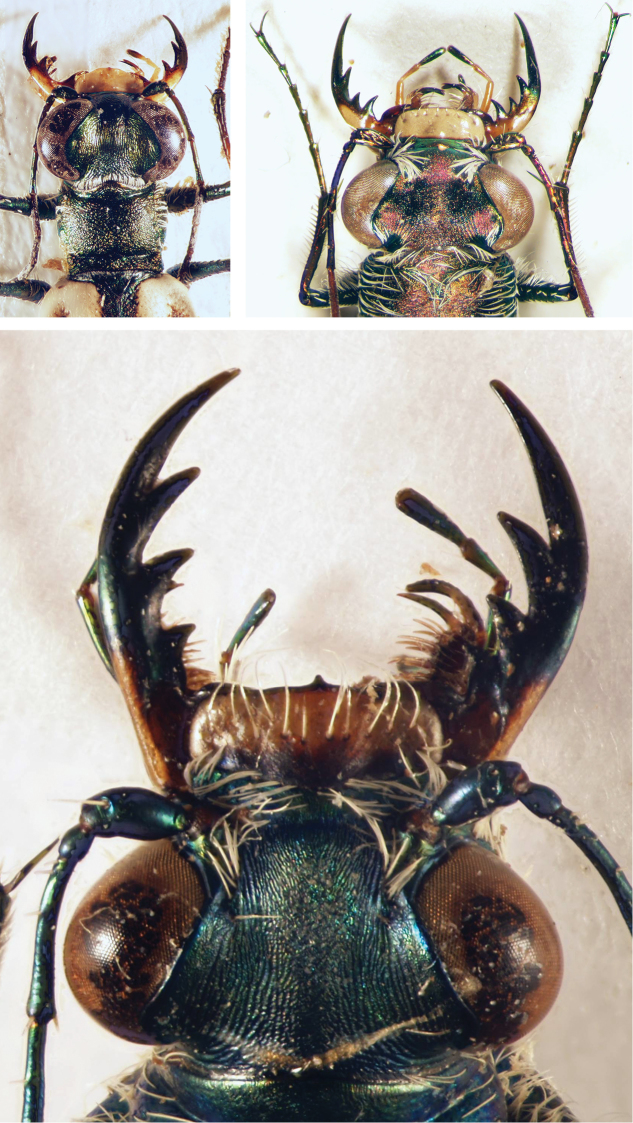
Forebody of cicindelid species: *Hypaetha singularis* (above, left), *Habrodera nilotica* (above, right), and *Calomera alboguttata* (below).

**Figure 56.**
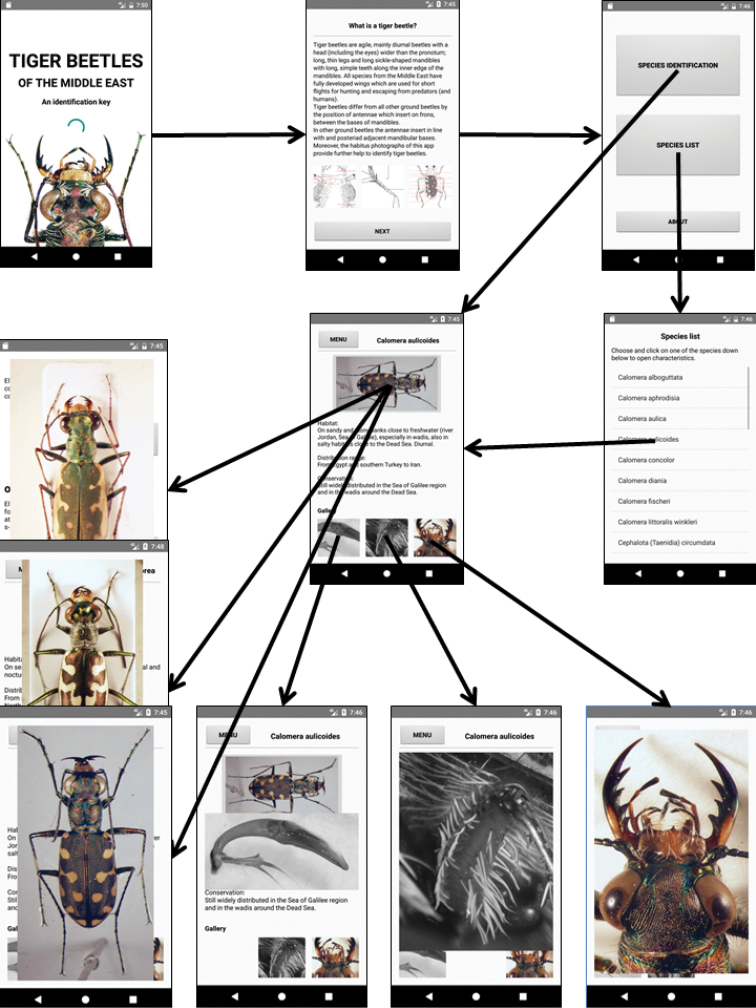
Overview of the main functions of the application TIGER BEETLES ID. This stand-alone application for mobile Android devices (smartphones, tablets) can be freely downloaded at https://doi.org/10.3897/zookeys.734.21989.suppl1.

##### Distribution range.

From Egypt east of the Nile to the Arabian Desert (Oman, Yemen), also found in tropical Africa (Werner 2000; Wiesner 1992).

##### Distribution in the southern Levant.

Only in southern Sinai (Abdel-Dayem et al. 2003; Nussbaum 1987), from where the SMNHTAU records also originate (Matalin and Chikatunov 2016). Ptashkovsky (2013) recorded the species for Israel, but no verifiable records are found in SMNHTAU, which includes the collection of Ptashkovsky.

#### 26. Lophyra flexuosa

Taxon classificationAnimaliaColeopteraCicindelidae

(Fabricius, 1787)

##### Habitat.

Eurytopic species found on sea shores, in saltmarshes, in dune depressions, on river banks, in oases and in palm plantations, not restricted to coastal habitats (Abdel-Dayem et al. 2003; Jaskuła and Rewicz 2015; Lisa 2002; Nussbaum 1987).

##### Phenology.

Throughout most of the year, from February to December (Matalin and Chikatunov 2016; Nussbaum 1987).

##### Distribution range.

From Morocco to Israel (Wiesner 1992).

##### Distribution in the southern Levant.

Numerous records from the Mediterranean coast in Israel and northern Sinai, southwards to the Negev and central Sinai (Abdel-Dayem et al. 2003; Horn 1931; Matalin and Chikatunov 2016; Nussbaum 1987).

##### Conservation.

Not threatened. A widespread species which also can be found in highly disturbed habitats.

#### 27. Lophyra hilariola

Taxon classificationAnimaliaColeopteraCicindelidae

(Bates, 1874)

##### Habitat.

On sparsely vegetated escarpments along rivers (Franzen and Bischoff 1995).

##### Phenology.

Poorly known, records from April and May, but may have a longer activity period (Franzen and Bischoff 1995).

##### Distribution range.

From Turkey to Iran (Wiesner 1988).

##### Distribution in the southern Levant.

No record.

##### Taxonomic notes.

A table found in Franzen and Bischoff (1995) can be used to differentiate *L. hilariola* from *L. flexuosa*.

#### 28. Lophyra histrio

Taxon classificationAnimaliaColeopteraCicindelidae

(Tschitschérine, 1903)

##### Habitat.

On beaches, in salt marshlands and in freshwater habitats; can be found together with *C. fischeri* (Wiesner 1996).

##### Phenology.

February to September (Wiesner 1996).

##### Distribution range.

From the Arabian Peninsula to India (Wiesner 1992).

##### Distribution in the southern Levant.

No record.

### Compilation of the distribution of the tiger beetles in the southern Levant and adjacent lands

Verifiable records are ascertained for 14 species from the southern Levant, as 10 of them live in Israel, 10 occur in the Sinai and 4 live in Jordan. From the adjacent countries, 20 additional species have been recorded (Table 1).

**Table 1.** The tiger beetle species of the southern Levant (Israel, Jordan, Sinai) and adjacent areas of the neighboring countries (Egypt west of the Nile, western Iraq, Lebanon, northern Saudi Arabia, Syria without its eastern parts). Species with numbers larger than 29 are not mentioned in the keys and the species accounts. V: vulnerable, E: endangered, CE: critically endangered or extinct. X: species with verifiable record(s), data deficient for a threatened category or not threatened. (X): species found in Egypt, Syria, Iraq and/or Saudi Arabia, but outside the range of the identification keys. No: listed, but no verifiable records from the given country, probably misidentified. – : no record and not listed.

Species	Egypt (Sinai)	Israel	Jordan	Adjacent countries
1. *Grammognatha euphratica* (Dejean, 1822)	X	E	X	X
2. *Cicindela javetii* Chaudoir, 1861	–	CE	–	X
3. *Cicindela herbacea* Klug, 1832	–	–	–	X
4. *Cicindela asiatica* Audouin & Brullé, 1839	–	–	–	X
5. *Calomera concolor* (Dejean, 1822)	–	–	–	X
6. *Calomera fischeri* (Adams, 1817)	–	No	No	(X)
7. *Calomera alboguttata* (Klug, 1832)	–	–	–	X
8. *Calomera aulica* (Dejean, 1831)	X	E	X	X
9. *Calomera diania* (Tschitschérine, 1903)	–	–	–	(X)
10. *Calomera aphrodisia* (Baudi di Selve, 1864)	–	X	–	X
11. Calomera littoralis (Fabricius, 1787) , ssp. winkleri (Mandl, 1934)	–	X	–	X
12. *Calomera aulicoides* (J.R. Sahlberg, 1913), stat. rest.	X	X	X	X
13. *Calomera fimbriata* (Dejean, 1831)	–	–	–	(X)
14. *Habrodera nilotica* (Dejean, 1825)	X	No	–	X
15. Cephalota (Taenidia) littorea (Forskål, 1775)	X	–	–	X
16. Cephalota (Taenidia) tibialis (Dejean, 1822)	X	–	–	X
17. Cephalota (Taenidia) circumdata (Dejean, 1822)	No	–	–	–
18. Cephalota (Taenidia) vartianorum (Mandl, 1967)	–	CE	–	X
19. *Homodela ismenia* (Gory, 1883)	–	–	–	X
20. Cylindera (Eugrapha) contorta (Fischer von Waldheim, 1828) , ssp. valdenbergi (Mandl, 1981)	X	CE	–	–
21. Cylindera (Ifasina) rectangularis (Klug, 1832)	–	–	–	(X)
22. Myriochila (s.str.) melancholica (Fabricius, 1798)	X	X	X	X
23. Myriochila (Molenica) orientalis (Dejean, 1825)	–	–	–	X
24. Myriochila (Molenica) dorsata (Brullé, 1834)	No	–	–	(X)
25. *Hypaetha singularis* (Chaudoir, 1876)	X	No	–	X
26. *Lophyra flexuosa* (Fabricius, 1787)	X	X	–	X
27. *Lophyra hilariola* (Bates, 1874)	–	–	–	(X)
28. *Lophyra histrio* (Tschitschérine, 1903)	–	–	–	(X)
29. *Cephalota deserticola* (Faldermann, 1836)	–	No	–	–
30. *Cylindera pygmaea* (Dejean, 1825)	–	–	–	(X)
31. *Calomera caucasica* (Adams, 1817)	–	–	–	(X)
32. *Salpingophora bellana* (W. Horn, 1905)	–	–	–	(X)
33. *Salpingophora hanseatica* (W. Horn, 1927)	–	–	–	(X)
34. *Salpingophora rueppelii* (Guérin-Méneville, 1847)	–	–	–	(X)
35. *Hypaetha schmidti* (W. Horn, 1927)	–	–	–	(X)
36. *Hypaetha copulata* (Schmidt-Göbel, 1846)	–	–	–	(X)

Three species are listed from the southern Levant, but their occurrence is questionable as verifiable records are missing: *Calomera fischeri, Cephalota circumdata* and *Cephalota deserticola*.

## Discussion

### Identification tools

We present two formats of the same key which enables the identification of the tiger beetles of Egypt, western Iraq, Israel (including the areas under Palestinian control), Jordan, Lebanon, Syria (without the easternmost parts) and northern Saudi Arabia. In the investigated study region, the southern Levant, there are Geadephaga species for which poleward shifts in their distribution ranges due to global change have been identified (e.g. Drees et al. 2011). The incorporation of the southern areas in our study may ease the identification of comparable shifts in tiger beetles.

Under laboratory conditions, all species can be reliably identified using the “classical” identification key, including those requiring the dissection of the male genitalia (e.g. sibling species *Cicindela javetii*, *C. herbacea*; *Calomera aulicoides*, *C. littoralis*, and *C. aulica*). However, the majority of the species can be identified correctly under field conditions, by examining live individuals using basic magnification tools. In such circumstances the Android application may be more useful rather than the classic key. We hope that both identification tools will be useful in a range of contexts, such as education at levels, academic research, the activities of citizen scientists and in practical conservation work like surveying.

Our application for mobile Android devices, TIGER BEETLES ID, can serve as a starting point for the development of additional tools, with the translation of the app’s text into both Hebrew and Arabic being greatly desired. Moreover, a simple version of the identification application is possible by deleting those alternatives considering the species which occur exclusively outside of Israel, the areas under Palestinian control, and Jordan. The simplified version may better address the need of less experienced users such as biology teachers at secondary schools and their students. For this version, appropriate translations of morphological terms (for example genae, palpi, etc.) must be taken into consideration, and in some cases may need to be developed, as established terms in Hebrew and in Arabic are in many cases lacking.

### Faunistic inventory of the tiger beetles of the southern Levant

As far as we know, the first record of *Calomera aphrodisia* for Israel has now been documented in the form of an old specimen in ZISP. The occurrence of two species is confirmed by new records from Jordan (*Grammognatha euphratica* and *Calomera aulica*). It is likely that additional species which occur in Jordan have not yet been recorded (e.g., *Cephalota vartianorum* in the vicinity of the Dead Sea, see also below), especially as the oases in the eastern part of the country have, to our knowledge, not yet been sampled.

In terms of tiger beetle faunistics, Israel is certainly the best-studied country in the Middle East, as shown by the number of records found in SMNHTAU which are listed by Matalin and Chikatunov (2016). However, here too, we list one first record for Israel (see above) as well as first local records (for example *Calomera aulicoides* for the northern Hula Valley, *Calomera littoralis* in eastern Lower Galilee, *Calomera aulica* on the Mediterranean Sea coast). The material found in the collection of the Steinhardt Museum at the Tel Aviv University (SMNHTAU) can be used to help bridging the gap between taxonomy and nature conservation biology. Thus the knowledge of the distribution of tiger beetles can be deepened, and the decline of many tiger beetle species can be investigated. This is of particular importance in regions such as the southern Levant, where species diversity has not yet been thoroughly studied in terms of taxonomy and systematics (cf. Braby and Williams 2016). Additional intensive sampling, especially of protected areas, is needed across the region.

### Conservation biology of tiger beetles in the southern Levant

For the classification of threatened species, we used an approach which is widely used in Central Europe (Ludwig et al. 2006; Seibold et al. 2015). In general, threatened species are classified following the IUCN criteria for Red Lists (IUCN 2004; 2017). However, these criteria are sometimes criticized, especially for the classification of insects (e.g. Braby and Williams 2016). To allow for more convenient comparison with vertebrates and plants, we plan to apply the IUCN criteria in a future publication.

Five tiger beetle species have been classified as threatened. Two species, *Grammognatha euphratica* and *Calomera aulica*, are defined as vulnerable, meaning that they have become rare in Israel, and probably in Jordan as well. However, both taxa are widely distributed outside of the southern Levant and seem not to be threatened in other parts of their distribution ranges. *Grammognatha euphratica* is apparently expanding its distribution range northwards (Cassola et al. 2014), perhaps as result of climate change.

Three critically endangered species have not been recorded in Israel during the last decade. Matalin and Chikatunov (2016) stated that there are no records for these species since the 1980s or 1990s. Despite the existence of additional, more recent records (see above), the populations of these species are clearly in decline, and they are most likely very rare, already extinct, or close to extinction, at least in Israel. The three species are:

(a) *Cephalota vartianorum*, for which apparently suitable habitats remain in the salt marshes on both the Israeli and on the Jordanian sides of the Dead Sea. However, this species seems to have disappeared from nature reserves where it was recorded in 1994 (e.g. Enot Zuqim), as we did not find individuals in any of our collecting trips, including nocturnal light trapping methods. Specifically in Enot Zuqim, a decline of the phytodiversity in this nature reserve has been reported (Olsvig-Whittaker et al. 2009).

(b) The known habitat of *Cicindela javetii* in the Golan Heights has been destroyed, and no further records since the 1990s are known from Israel. Due to the high power of dispersal of the species, with all individuals being fully winged and flight-active, (re-) colonization of suitable habitats in the southern Levant is possible.

(c) Israel and Egypt are responsible for the world-wide preservation of *Cylindera contorta valdenbergi*, as its entire distribution range is confined to these two countries. However, it is unclear if populations of this taxon still exist, or if *C. contorta valdenbergi* is extinct. The definition of national responsibility is important in the assessment of national conservation priorities as well as in decision making about inclusion in international conventions on species conservation. The larval development of *Cylindera contorta valdenbergi* occurs in close proximity to sea water line on beaches, a habitat which is often extremely disturbed by tourism and recreational activities such as swimming facilities and off-road vehicles which compress the soil and sand and destroy the habitat of the species. For *Habroscelimorpha dorsalis* s.str. Say (= *Cicindela dorsalis* s.str.), an endangered species covered by the U.S. Endangered Species Act (Knisley et al. 1998) which inhabits similar habitats in North America, such vehicles have been identified as the main cause in the species’ decline (Knisley et al. 2016). We suggest a thorough survey of all near-natural beach sections between Gaza Strip and Akko, particularly where *C. contorta valdenbergi* has previously been recorded, in order to ascertain whether any population of this taxon still exists. Such a survey can serve as the basis for the development of conservation policy and as a baseline for future monitoring of population sizes. Relevant methods developed for *H. dorsalis* can be found in Knisley et al. (2016).

We hope that our identification tools and the species’ accounts describing the ecology and conservation biology of the tiger beetles of the southern Levant will encourage further work on tiger beetles in the Middle East and enhance the conservation and preservation of these attractive insects.

## Supplementary Material

XML Treatment for Grammognatha euphratica

XML Treatment for Cicindela javetii

XML Treatment for Cicindela herbacea

XML Treatment for Cicindela asiatica

XML Treatment for Calomera concolor

XML Treatment for Calomera fischeri

XML Treatment for Calomera alboguttata

XML Treatment for Calomera aulica

XML Treatment for Calomera diania

XML Treatment for Calomera aphrodisia

XML Treatment for Calomera littoralis (Fabricius, 1787) , ssp. winkleri

XML Treatment for Calomera aulicoides

XML Treatment for Calomera fimbriata

XML Treatment for Habrodera nilotica

XML Treatment for Cephalota (Taenidia) littorea

XML Treatment for Cephalota (Taenidia) tibialis

XML Treatment for Cephalota (Taenidia) circumdata

XML Treatment for Cephalota (Taenidia) vartianorum

XML Treatment for Homodela ismenia

XML Treatment for Cylindera (Eugrapha) contorta (Fischer von Waldheim, 1828) , ssp. valdenbergi

XML Treatment for Cylindera (Ifasina) rectangularis

XML Treatment for Myriochila (s.str.) melancholica

XML Treatment for Myriochila (Monelica) orientalis

XML Treatment for Myriochila (Monelica) dorsata

XML Treatment for Hypaetha singularis

XML Treatment for Lophyra flexuosa

XML Treatment for Lophyra hilariola

XML Treatment for Lophyra histrio

## Supplementary materials

Supplementary material 1 TIGER BEETLE ID Data type: application (apk)
*Disclaimer of warranties.* You expressly understand and agree that your use of the app “TIGER BEETLES ID” is at your sole risk and that the app is provided “as is” and “as available” without warranty of any kind from the authors. Your use of the app and any material downloaded or otherwise obtained through the use of the app is at your own discretion and risk, and you are solely responsible for any damage to your computer system or other device or loss of data that results from such use. The authors further expressly disclaim all warranties and conditions of any kind, whether expressed or implied, including, but not limited to the implied warranties and conditions of merchantability, fitness for a particular purpose and non-infringement. This is an open access article distributed under the terms of the Creative Commons Attribution License (CC BY 4.0), which permits unrestricted use, distribution, and reproduction in any medium, provided the original author and source are credited.Thorsten Assmann, Estève Boutaud, Jörn Buse, Jörg Gebert, Claudia Drees, Ariel-Leib-Leonid Friedman, Fares Khoury, Tamar Marcus, Eylon Orbach, Ittai Renan, Constantin Schmidt, Pascale Zumstein
